# Multivalent Presentation of MPL by Porous Silicon Microparticles Favors T Helper 1 Polarization Enhancing the Anti-Tumor Efficacy of Doxorubicin Nanoliposomes

**DOI:** 10.1371/journal.pone.0094703

**Published:** 2014-04-15

**Authors:** Ismail M. Meraz, Claire H. Hearnden, Xuewu Liu, Marie Yang, Laura Williams, David J. Savage, Jianhua Gu, Jessica R. Rhudy, Kenji Yokoi, Ed C. Lavelle, Rita E. Serda

**Affiliations:** 1 Nanomedicine, Houston Methodist Research Institute, Houston, Texas, United States of America; 2 Adjuvant Research Group, School of Biochemistry and Immunology, Trinity Biomedical Sciences Institute, Trinity College, Dublin, Ireland; 3 Advanced Materials and BioEngineering Research (AMBER), Centre for Research on Adaptive Nanostructures and Nanodevices (CRANN), Trinity College, Dublin, Ireland; 4 Michael E. DeBakey Department of Surgery, Baylor College of Medicine, Houston, Texas, United States of America; 5 The University of Texas School of Medicine, Houston, Texas, United States of America; Université Libre de Bruxelles, Belgium

## Abstract

Porous silicon (pSi) microparticles, in diverse sizes and shapes, can be functionalized to present pathogen-associated molecular patterns that activate dendritic cells. Intraperitoneal injection of MPL-adsorbed pSi microparticles, in contrast to free MPL, resulted in the induction of local inflammation, reflected in the recruitment of neutrophils, eosinophils and proinflammatory monocytes, and the depletion of resident macrophages and mast cells at the injection site. Injection of microparticle-bound MPL resulted in enhanced secretion of the T helper 1 associated cytokines IFN-γ and TNF-α by peritoneal exudate and lymph node cells in response to secondary stimuli while decreasing the anti-inflammatory cytokine IL-10. MPL-pSi microparticles independently exhibited anti-tumor effects and enhanced tumor suppression by low dose doxorubicin nanoliposomes. Intravascular injection of the MPL-bound microparticles increased serum IL-1β levels, which was blocked by the IL-1 receptor antagonist Anakinra. The microparticles also potentiated tumor infiltration by dendritic cells, cytotoxic T lymphocytes, and F4/80^+^ macrophages, however, a specific reduction was observed in CD204^+^ macrophages.

## Introduction

The physico-chemical properties of synthetic particles have a major impact on their cellular associations, biodistribution, degradation, and immuno-modulatory properties. Alum, an aluminum salt particulate used in vaccines, is thought to recruit immune cells to the site of injection, at least in part due to cytotoxic effects. [1] While macrophages internalize alum, dendritic cells (DC) are reported to exhibit no or limited uptake of alum. [2] Alum favors T helper (Th)2 immune responses and does not stimulate production of proinflammatory Th1 promoting cytokines by DCs or macrophages. [3]–[5] However, effective cancer immunotherapies require Th1 cytokines to arrest tumor growth, specifically interferon (IFN)-γ and tumor necrosis factor (TNF)-α. [6] To overcome this deficiency, a novel formulation containing alum and the Toll-like receptor (TLR)-4 ligand monophosphoryl lipid A (MPL) has been used to enhance adaptive immunity. [7] MPL, which is approved for clinical use in vaccines, induces secretion of IL-12 by antigen presenting cells (APC), leading to IFN-γ secretion by T and NK cells [8], [9].

The NOD-like receptor family, pyrin domain containing 3 (NLRP3) inflammasome is a multiprotein complex that functions to activate caspase-1, leading to the proteolytic maturation and secretion of the proinflammatory cytokines interleukin (IL)-1β and IL-18. [10] Chemotherapeutics, such as doxorubicin, activate the NLRP3 inflammasome, and mice lacking components of the NLRP3 inflammasome fail to respond to chemotherapy unless exogenous IL-1β is provided [11].

As an alternative to alum, synthetic particles have the potential to support Th1 responses in the absence of cytotoxicity based on intrinsic particle properties, as well as through presentation of pathogen-associated molecular patterns or proinflammatory cytokines. In 2008, Eicke Latz *et al.* [12], [13] reported that silica crystals activate the NALP3 inflammasome in PBMCs in a process dependent on phagocytosis. We previously demonstrated [14] that porous silicon (pSi) microparticles enhance lipopolysaccharide (LPS) and MPL mediated DC secretion of proinflammatory cytokines that are associated with successful cancer immunosurveillance. [15] Direct attachment of the molecule to the particle surface generated a multivalent ligand for TLR-4, with superior activation of downstream pathways compared to free MPL or LPS leading to secretion of IL-1β and proinflammatory cytokines such as TNF-α. *In vitro*, pSi microparticle stimulated IL-1β secretion was caspase-dependent and slightly elevated in the presence of cytochalasin B, [14] which is known to induce frustrated phagocytosis, locking silicon microparticles on the cell surface in actin rings, thereby inducing sustained engagement of surface receptors. [16] pSi presentation of adsorbed MPL led to increased particle uptake by DC; elevated DC expression of costimulatory and major histocompatibility complex (MHC) class I and II molecules; stimulated migration of DC to the draining lymph node; and enhanced associations between DCs presenting the ovalbumin peptide SIINFEKL and T cells from C57BL/6-Tg(TcraTcrb) (OT-1) transgenic mice [14].

Intravenous injection of oxidized (anionic) or cationic pSi microparticles in mice has been shown to be inert with respect to endothelial cell cycle, mitosis [17] and *in vivo* tumor cell growth. [18], [19] The biodistribution and biodegradability of pSi microparticles is well documented. [20]–[22] Functionally, pSi microparticles are proven carriers for nanoparticles, nucleic acids, peptides, and chemotherapeutic agents [23], [24].

In this study, for the first time we examine in depth the *in vivo* immunomodulatory properties of pSi microparticles and the impact of multivalent presentation of MPL, as well as the combined potential of these agents to augment the immunomodulatory properties of chemotherapeutics. Using an immune competent mouse model of breast cancer, pSi microparticles in combination with doxorubicin-loaded nanoparticles (DOX-NPs) are administered by intravenous injection. In addition, the impact of intraperitoneal injection of microparticles of various sizes and aspect ratios are examined to explore the impact of geometry and size on intrinsic immunomodulatory properties. Cell recruitment to the site of injection and to the tumor microenvironment is explored, as is the impact of the microparticles on the cytokine milieu. MPL-adsorbed pSi microparticles are found to favor Th1 polarization in the tumor and have anti-tumor properties, independent of and additive to that induced by DOX-NPs.

## Materials and Methods

### Ethics Statement

All animal procedures were performed in accordance with protocols reviewed and approved by the Institutional Animal Care and Use Committee at Houston Methodist and Trinity College as appropriate.

### Materials

LPS from *Escherichia coli* and MPL from *Salmonella enteric* serotype Minnesota RE 595 were purchased from Sigma-Aldrich (St. Louis, MO, USA). IL-1β, TNF-α and IFN-γ ELISA kits were purchased from R&D Systems, Inc. (Minneapolis, MN, USA; detection range 15.6–1000 pg/ml, 31.2–2000 pg/ml and 31.2–2000 pg/ml respectively). IL-10 ELISA kits were purchased from Biolegend, Inc. (San Diego, CA, USA; detection range of 31.3–2000 pg/ml). IL-12p40 capture (purified) and detection (biotin) antibodies were purchased from BD Pharmingen; detection range of 39.06–5000 pg/ml). If samples reached the top end of the standard curve for ELISA assays, samples were diluted 1∶2 for more accurate readings. Alum was purchased from Brenntag Biosector (Alhydrogel). Liposomal Doxorubicin (DOX-N), 80–90 nm, was purchased from Avanti Polar Lipids, Inc. (Alabaster, AL, USA). 4T1-luc2-td Tomato Bioware Ultra Red mouse mammary cancer cells were purchased from Caliper Life Sciences (Hopkinton, MA, USA). Human THP-1 monocytes were purchased from American Type Culture Collection (ATCC; Manassas, VA, USA).

### Animals Models

BALB/c (6–8 wk old) mice were obtained from Charles River Laboratories, Inc, Wilmington, MA, USA) and C57BL/6 mice from The Jackson Laboratory (Bar Harbor, ME, USA). NLRP3^−/−^ mice were generously provided by Professor Jurg Tschopp and bred in the Trinity College Dublin Bioresources Unit.

### Particle Fabrication

Starting with a p-type <100> wafer with resistivity of 0.005 ohm-cm as the substrate (Silicon Quest, Inc, Santa Clara, CA, USA), the wafer was exposed to 1∶3 hydrofluoric acid (49%):ethanol solution, and electrochemically etched with a DC current density of 7 mA/cm^2^ for 65 sec and 125 sec to form 200 and 400 nm thick porous silicon layers, respectively. The current density was then increased to 76 mA/cm^2^, and kept for six sec to form a second porous layer, the high porosity release layer. The bi-layer porous films were washed and spin-dried. An 80 nm thick low-temperature oxide (LTO) was deposited on the porous film in a LPCVD furnace at 400°C. The photolithography process was then carried out using an i-line contact aligner (KARL SUSS MA6 mask aligner). To make 1800×400 nm rod-shape microparticles, a dark-field photomask (chrome on quartz; HTA Photomask) consisting of a pattern array of 1800×400 nm rectangles with 2500 nm×1000 nm pitch was used. A thin NR9-250P photoresist (Futurrex Franklin, NJ, USA) layer was spun on the double-layer film at 3000 RPM and baked. The resist exposure time and development process were optimized to obtain clean 1800×400 nm patterns on 400 nm thick film. Discoidal microparticles with 2500, 1000 and 600 nm diameters were patterned on the 200 or 400 nm thick porous silicon films using NR9-500P photoresist (Futurrex Franklin, NJ, USA) and the dark-field photomasks consisting of arrays of circles with 2600, 1100 and 600 nm diameters, respectively. Both rod-shaped and discoidal patterns were transferred into the double-layer porous films by reactive ion etch in CF4 plasma (Plasmatherm BatchTop, 15 sccm CF4, 100 mTorr, 200 W RF). After striping the LTO in dilute HF, the as-made porous silicon particles were retained on the substrate allowing go through washing steps, followed by release using ultrasound in isopropyl alcohol. Particles were oxidized in a 35% H_2_O_2_ solution at 100°C for two hr.

Microparticles were treated with piranha solution (1 volume H_2_O_2_ and 2 volumes of H_2_SO_4_) to chemically oxidize the surface. The suspension was heated to 110–120°C for two hr, centrifuged and washed in deionized water repeatedly. Amine surface modification was achieved using a 2% (v/v) 3-aminopropyl triethoxy-silane (APTES) (Sigma-Aldrich) solution in 95% isopropanol and 5% water for two hr at 35°C with agitation at 1300 RPM.

MPL was adsorbed to pSi microparticles by immersion in a 1 mg/ml aqueous solution of MPL followed by two wash steps. The binding density of MPL was determined by imaging using a Bruker Multimode Atomic Force Microscope as described previously and the amount of bound MPL was determined by Western blot analysis or quantitating fluorescent ligand bound to the microparticles [14].

Cationic MPL liposomes were made by dissolving lipids and cholesterol at a molar ratio of 7∶3:1 DPPC:Cholesterol:DOTAP in a 3∶1 solution of chloroform and methanol. MPL (250 µg per 40 mg lipid) was added from a Chloroform: Methanol (3∶1) stock solution. Solvent was extracted at 55°C using a Hei-Vap Series Heidolph Rotary Evaporator (Heidolph Instruments GmbH & Co., Schwabach, Germany). PBS was added and the mixture was heated at 52°C for three min, vortexed for 2.5 min, and then sonicated for 30 sec with three repeats. A Malvern Zetasizer (Worcestershire, UK) was used to measure particle size and surface potential.

### Preparation of Bone Marrow-derived Dendritic Cells (BMDC)

BMDCs were prepared as previously described. [14] Briefly, bone marrow cells were harvested from the femurs and tibias of mice and filtered through a cell strainer (BD Biosciences, San Jose, CA, USA). RBC was lysed with ACK lysis buffer (Quality Biologicals, Inc., Gaithersburg, MD, USA), and cells were washed and cultured in six-well plates at 10^6^ cells/ml (3 ml/well) in RPMI 1640 (Sigma-Aldrich) supplemented with 10% fetal bovine serum (FBS) (Atlanta Biologicals, Lawrenceville, GA, USA), 100 U/ml penicillin/streptomycin (Fishers Scientific, Waltham, MA, USA), 50 µM 2-mercaptoethanol (Sigma-Aldrich), and mouse granulocyte-macrophage colony stimulating factor (GM-CSF) (20 ng/ml; Pierce, Rockford, IL, USA) plus or minus IL-4 (100 ng/ml) as indicated for 7–10 days. Half of the media was replaced every 2–3 days with fresh media and cytokines.

### Imaging Microparticle Uptake by BMDC using Electron Microscopy

BMDC cells were plated in 24 well plates containing 5×7 mm silicon chip specimen supports (Ted Pella, Inc., Redding, CA, USA) at 1×10^5^ cells per well. The next day, cells were incubated with pSi microparticles for one hr at 37°C at a 1∶20 cell to microparticle ratio. Cells were processed for SEM imaging as previously described.^14a, 16^ Specimens were mounted on SEM stubs (Ted Pella, Inc.) using conductive adhesive tape (12 mm OD PELCO Tabs, Ted Pella, Inc.), and sputter-coated with a four nm layer of platinum/palladium (80∶20) using a Cressington Sputter Coater 208 HR (Ted Pella, Inc.). For microparticle imaging, pSi microparticles in IPA were dried on SEM stubs. SEM images were acquired under high vacuum, at 10–15 kV, spot size 3.0, using a Nova NanoSEM Scanning Electron Microscope (FEI Company, Hillsboro, OR, USA). Gamma levels of the micrographs were adjusted to enhance image contrast and brightness.

For transmission electron microscopy, GM-CSF-stimulated BMDC were cultured on poly-l-lysine glass slides, and then incubated with TLR ligand-bound pSi microparticles (1∶20 ratio) for four hr followed by washing and fixation in 2% glutaraldehyde in 0.1 M cacodylate buffer, pH 7.4. After washing with cacodylate buffer, cells were incubated in a mixture of osmium tetroxide and 1% potassium ferrocyanate in 0.1 M cacodylate buffer for 30 min at 48°C. Cells were then dehydrated in increasing concentrations of ethanol and embedded with a mixture of epon and araldite. After polymerization at 60°C, glass slides were removed from the resin by freezing in liquid nitrogen. Ultrathin sections were counter-stained with uranyl acetate and imaged using a JEOL 1210 microscope equipped with an AMT Imaging System (JEOL, Peabody, MA, USA). Gamma adjustments were made to the micrographs to enhance image contrast and brightness.

Alum uptake by BMDC (GM-CSF and IL-4 culture; five days) was examined by transmission electron microscopy following treatment with 2 µg/ml Imject alum (Thermo Scientific) for four hr. Cells were washed in PBS twice followed by fixation in TRUMPS fixative and processing as described in the preceding paragraph.

### Evaluation of Cell Viability

BMDCs were incubated with three doses of alum (25, 12.5, and 6.25 µg/ml), LPS (10 ng/ml), or pSi microparticles at a dose of 20 microparticles/cell for 24 hr. Cells were washed and stained with Aqua Live/Dead and evaluated using flow cytometry. Samples were acquired using Summit software (Dako, Colorado, USA) and the data were analysed using FlowJo software (Treestar, Oregon, USA).

### pSi Microparticle-mediated Leukocyte Infiltration

Female mice (C57BL/6) were injected intraperitoneally with either PBS, Alum (0.3 mg/mouse), 0.3 mg pSi microparticles, MPL (50 µg/mouse) or pSi particles loaded with MPL. After 24 hr the mice were euthanized by CO_2_, the peritoneal cavity was washed with PBS and the peritoneal extrudate cells (PEC) were harvested for flow cytometry analysis and/or *ex vivo* restimulation. For restimulation experiments, PECs were plated at 1×10^6^ cells/ml and restimulated with either CpG (5 µg/ml) or Heat-killed E. coli (10 bacteria:1 cell) for 24 hr. Supernatants were collected and IFN-γ, TNF-α, IL-12p40 and IL-10 concentrations were determined by ELISA.

For flow cytometry analysis, PECs (1×10^6^ cells/ml) were incubated for 30 min with AQUA fluorescent dye (0.5 µl) (Invitrogen) and for an additional 30 min with antibodies specific for CD11b (0.005 µg) (M1/70 : BD Pharmingen), Gr-1 (0.04 µg) (RB6-8C5 : BD Pharmingen), F4/80 (0.1 µg) (BM8 : eBioscience), ckit (0.5 µg) (2B8 : BD Pharmingen), CD11c (0.1 µg) (HL3 : BD Pharmingen), MHCcII (0.04 µg) (M5/114.15.2 : BD Pharmingen), CD19 (0.1 µg) (1D3 : BD Pharmingen), Ly6C (0.25 µg) (AL-21 : BD Pharmingen) and SiglecF (0.02 µg) (E50-2440 : BD Pharmingen). Samples were acquired using a Becton Dickinson FACSCanto flow cytometer equipped with Summit software (Dako, Colorado, USA) and the data was analysed using FlowJo^T^ software (Treestar, Oregon, USA).

### Therapeutic Efficacy Studies

Breast cancer tumors were established in BALB/c mice by intramammary injection of 1×10^5^ 4T1 cells. On day 10–14, mice were inoculated weekly by intravenous tail vein injection as follows: PBS control, DOX-NP (2.5 or 5.0 mg/kg); MPL (10 µg); MPL-pSi microparticles (5×10^8^); or the hybrid combination of DOX-NP/MPL-pSi particles. Alternatively, mice were injected intratumorally with PBS, MPL (10 µg), or MPL-liposomes (1000 µg lipid/6.25 µg MPL; 50 µl). Kineret (anakinra, 30 mg/dose; Amgen, Thousand Oaks, CA, USA) was administered 3 times per week by intraperitoneal injection. Tumor growth was monitored by caliper measurements 3× per week and based on luciferase expression weekly with the Xenogen IVIS-200 System (Perkin Elmer Inc., Waltham, MA, USA) following intra-peritoneal injection of 150 mg/kg RediJect D-Luciferin (Perkin Elmer Inc.). Mice were sacrificed 24–26 days after initiation of tumor growth, blood was collected by cardiac puncture, and tumor and spleen were collected for weight, size and immunohistochemical staining.

### Immunohistochemistry

Tissues were quick frozen in OCT (Tissue-Tek) and stored at −80°C. Tissue sections (10 µm) were fixed with ice-cold acetone for 15 min at −20°C and washed three times with PBS followed by blocking with 5% FBS. Fluorescence-labeled primary antibodies [e-flour 615 CD8 (clone 53-6.7; 1∶50), e-flour 570 ki67 (clone solA15; 1∶100; eBioscience, San Diego, CA, USA); FITC F4/80 (MCA497A488; 1∶100), Alexa Fluor 647 CD204 (MCA1322; 1∶50, AbD Serotec, Raleigh NC), Alexa Fluor 647 Gr-1 (clone RB6-8C5; 1∶100; Biolegend), FITC CD11b (clone M1/70, 1∶100; Biolegend); and 33D1 (1∶50, BD Biosciences, San Jose, CA, USA; secondary anti-rat IgG Alexa Fluor 647; Invitrogen)] were incubated with tissues overnight at 4°C in the presence of 5% FBS. Slides were then washed three times with PBS and mounted with ProLong Gold AntiFade with DAPI (Invitrogen). Images were taken using an A1 Nikon confocal microscope (Belmont, CA, USA) and percent positive cells were determined by manual counting or using Image J software (National Institute of Health) equipped with the ITCN (Image-based tool for counting nuclei) Plugin (Thomas Kuo and Jiyun Byun; Center for Bio-image Informatics at UC Santa Barbara, CA, USA) with analysis of 3–6 arbitrary regions representing randomly selected tissues from greater than one mouse per group.

### ELISA

Cytokines were measured by ELISA using kits purchased from R&D Systems (IFN-γ, TNF-α, and IL-1β), Biolegend (IL-10), and BD Pharmingen (IL-12p40) according to the manufacturer’s instructions. Briefly, 96-well high binding ELISA plates were coated with purified anti-IL-12p40 antibody (0.5 µg/ml) diluted with PBS at 4°C overnight. After three washes, non-specific binding was blocked using 1% BSA for two hr at room temperature. The standards and PEC samples were then added and the plate incubated for two hr at room temperature. After five washes, biotin anti-IL-12p40 antibody (1 µg/ml) was added for two hr, followed by HRP-conjugated streptavidin for 20 min. Absorbance was measured at 490 nm within 30 min of adding the stop solution. Mean absorbance was compared to a standard curve.

IL-1β secretion in plasma was measured by ELISA using the BD OptEIA kit (BD Biosciences, San Diego, CA, USA; detection limit 31.3–2000 pg/ml) according to the manufacturer’s instructions. Briefly, 96-well ELISA plates were coated with IL-1β antibody diluted with coating buffer at 4°C overnight. After three washes, non-specific binding was blocked using blocking buffer provided by the manufacturer for one hr at room temperature. The standards and plasma samples were then added and the plate incubated for two hr at room temperature. After five washes, mouse HRP conjugated anti-IgG antibody was added for an additional hr, followed by substrate solution for 30 min. Absorbance was measured at 450 nm within 30 min of adding the stop solution. Mean absorbance was compared to a standard curve.

Wildtype or NLRP3^−/−^ BMDCs were plated at 6.25×10^5^ cells/ml and primed with LPS at 1 or 10 ng/ml for one hr before pSi particles were added based on a dose of 40 microparticles/cell or on silicon content (0.006 mg/ml high dose). After 24 hr, supernatants were collected and IL-1β concentrations were determined by ELISA.

*In vivo* cytokine release was measured in BALB/c mice 4T1 tumors following subcutaneous injection of free MPL or 5×10^8^ control or MPL-pSi microparticles (10 µg MPL equivalents; n = 3/group). Blood samples were collected at various time points post injection via retinal orbital access and serum was prepared by centrifugation at 2500 g for 10 min. Serum samples were stored at −80°C and analyzed by ELISA.

### Statistics

Statistical analysis was performed using Graphpad Prism 5 software. The means for three or more groups were compared by one-way ANOVA. Where significant differences were found, the Tukey-Kramer multiple comparisons test was used to identify differences between individual groups. For differences in restimulation data, a two-way ANOVA was used, followed by a bonferroni posttest. Alternatively, statistical comparisons were performed using a two-sample, equal variance, two-tailed T test. Error bars in graphical presentations represent standard deviations.

## Results

### Fabrication of pSi Microparticles

**Figure 1:**
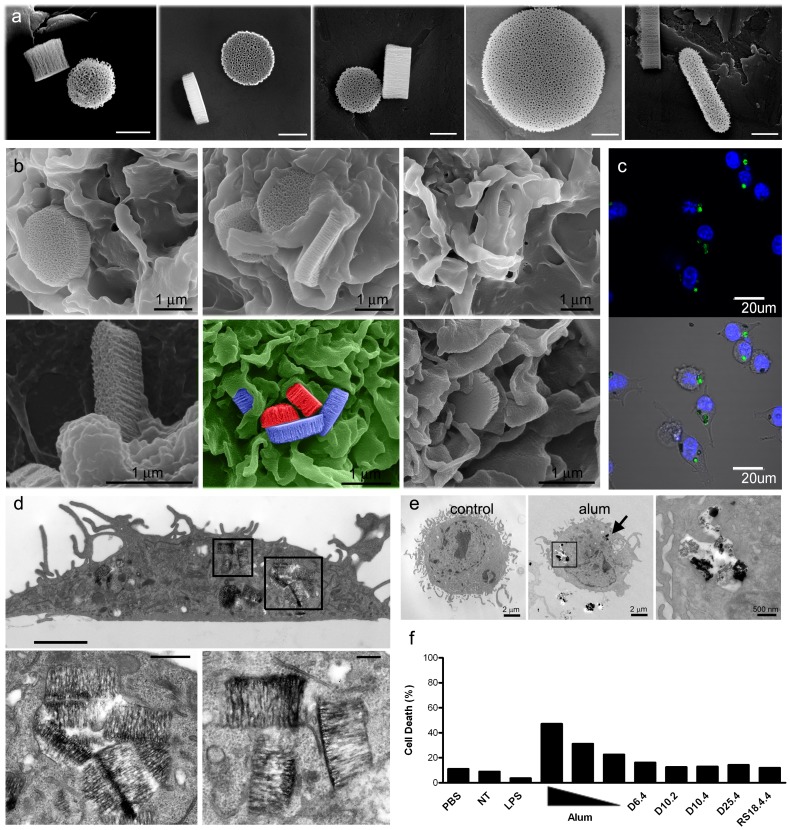
pSi microparticle association with BMDC and biocompatibility. a) Scanning electron micrographs show pSi microparticles of varying dimensions (bars 500 nm). b) SEM images show cellular association of 2500×400 nm pSi microparticles with BMDC at various stages of uptake (top row) and cellular association with rod-shaped (left, bottom row) and smaller discoidal particles [600 nm (middle) and 1000 nm (right)]. c) BMDCs, treated with five D25.4 microparticles/cell for three hr, were imaged using confocal microscopy. d) TEM images show BMDC with internalized TLR4-ligand (LPS) bound pSi microparticles four hr after microparticle introduction. Seven 1000×400 nm discoidal microparticles are seen in the cell in the upper image (boxed regions; bar 2 µm) and in the magnified regions below (bars 1 and 0.5 µm). e) TEM images show control (left) and alum-treated BMDC four hr after introduction of 2 µg/ml alum. The boxed region showing internalized alum is magnified in the image to the right. f) BMDCs were incubated with LPS (10 ng/ml), alum (25, 12.5 and 6.25 µg/ml), or pSi microparticles (20 particles/cell) for 24 hr. Cellular necrosis was evaluated using the LIVE/DEAD® Aqua dead cell stain.

Discoidal (D) and rod-shaped (R) pSi microparticles were fabricated by a process that consists of electrochemical etching of silicon films to create pores, photolithographic patterning of microparticle dimensions on pSi films, and reactive ion etch (RIE). As the porous structure and microparticle size are controlled independently, pSi microparticles with a wide range of dimensions, shapes and pore morphologies can be fabricated. Scanning electron micrographs of rod-shape and a variety of discoidal microparticles, with a mean pore size of 30 nm and a porosity of approximately 65%, are shown in **Figure 1a**. Particle lengths, widths, and abbreviations used in this study are presented in **Table 1**, along with a comparison of silicon content per microparticle number equivalents.

**Table 1:** Size and shape characteristics of pSi microparticles.

Abbreviation	Length (nm)	Width (nm)	Shape	mg Si/1e9 particles
D6.4	600	400	discoidal	0.11
D10.2	1000	200	discoidal	0.16
D10.4	1000	400	discoidal	0.3
D25.4	2500	400	discoidal	2.0
R18.4.4	1800	400×400	rod	0.3

The fabrication process began with a heavily doped silicon wafer. A bi-layer porous silicon film consisting of a device layer and a release layer was formed using step-current electrochemical etching in a hydrofluoric acid/ethanol solution. The release layer, with high porosity, was retained on the substrate, able to withstand extensive handling including flush and photoresist stripping, but collapsing rapidly upon sonication. The bi-layer porous film was then capped by an 80 nm low temperature oxide (LTO) deposited in a low pressure chemical vapor deposition furnace. A direct photolithographic process was followed to pattern the particle array on the LTO capped film. The particle patterns were transferred onto the bi-layer porous film by one-step RIE in CF4 plasma. After removing the residual photoresist and LTO layers, the resulting microparticles were retained on the wafer as an array. The monodispersed microparticles were obtained by ultrasonic treatment of the on-wafer microparticle array.

### BMDC Uptake of pSi Particles

To assess the impact of microparticle geometry and size on cellular interactions, various imaging modalities were used to capture various phases of microparticle uptake. Early uptake of the various microparticle formulations by BMDC induced membrane spreading across the microparticle surface that included actin cup formation and wrapping of microparticles in pseudopodia. Scanning electron micrographs shown in Figure 1b display cells one hr after addition of microparticles to the cell culture media. Further support for microparticle internalization by BMDC is presented in confocal micrographs three hr after microparticle addition (Figure 1c). Microparticles are visualized based on photoluminescence properties, while nuclei are labeled with Hoechst. Complete microparticle internalization and localization within phagosomes is presented in transmission electron micrographs of BMDC four hr after microparticle introduction (Figure 1d). Similar engulfment of alum (Imject) by BMDC is shown in electron micrographs in Figure 1e. BMDC were treated with 2 µg/ml alum for four hr. Internalization is apparent in two intracellular locations, with one region (boxed) shown at higher magnification in the far right image.

### Cytotoxicity of Adjuvants

Microparticle compatibility with primary BMDC was assessed using flow cytometry to measure cell viability. While treatment of BMDC with alum (25, 12.5, and 6.25 µg/ml; Brenntag Biosector) induced 22–49% cell death after 24 hr of incubation at 37°C, parallel exposure of BMDC to pSi caused no cell death above control levels at a dose of 20 particles per cell (Figure 1f). When microparticle toxicity was compared using equivalent doses of silicon across the spectrum, the largest microparticles (D25.4) displayed low levels of toxicity in a dose dependent manner (Figure S1).

### Impact of Size and Shape on pSi Microparticle Uptake by APC

**Figure 2:**
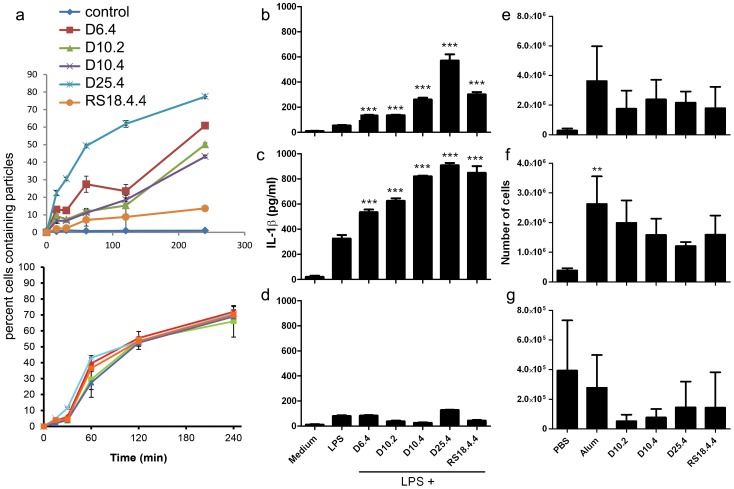
pSi microparticles are immunomodulatory both *in vitro* and *in vivo*. a) Fluorescent microparticle uptake by human monocytes (THP-1 cells) was measured by flow cytometry at select time points after adding microparticles to the cell culture media (top) or after settling of the microparticles on the cell surface by incubation of cells and microparticles on ice for 60 min (bottom) prior to adding warm media (37°C) and transferring to an incubator (n = 3/group). B-D) Wildtype (WT) (b, c) or NLRP3^−/−^ (d) BMDCs were primed with 1 ng/ml (b) or 10 ng/ml (c, d) LPS, followed one hr later with 40 particles/cell using pSi microparticles of varying geometries. After 24 hours, supernatants were collected and IL-1β concentrations were determined by ELISA (***p<0.001). E-G) Female C57BL/6 mice were injected i.p. with PBS, Alum or D10.2, D10.4, D25.4 and RS18.4.4 pSi microparticles (0.3 mg/mouse) and the mice were sacrificed after 24 hr. PECs were isolated and stained with various markers to identify cell populations in the peritoneum, specifically the number of neutrophils (e; CD11b^+^ Gr1^high^ F4/80^−^), eosinophils (f; Gr1^−^ ckit^−^ SiglecF^+^) and resident macrophages (g; CD11b^high^ F4/80^high^). **p<0.01 compared to PBS group, n = 3/group.

Microparticle uptake by antigen presenting macrophage-like (THP-1) cells varied by size and aspect ratio *in vitro* when microparticles were added to the culture media and time to settle on the cell surface was included in the kinetic analysis (**Figure 2a**). The largest microparticles (D25.4) had the greatest uptake across the four hr observation window, while the rod-shaped microparticles (RS18.4.4) displayed the lowest uptake. In contrast, uptake was similar for all microparticle sizes and shapes when contact with the cell surface was achieved prior to kinetic analysis by preincubation on ice.

### Synergistic Recruitment of Inflammatory Cells and Production of Proinflammatory Cytokines

To assess the ability of pSi microparticles to drive the secretion of the proinflammatory cytokine IL-1β, the effect of a broad range of pSi microparticle geometries on IL-1β production by BMDCs was assessed. All geometries of pSi microparticles induced significant IL-1β secretion from LPS-primed BMDCs, whether primed with 1 ng/ml (Figure 2b) or 10 ng/ml (Figure 2c) LPS. pSi microparticle mediated IL-1β secretion by BMDCs was NLRP3-dependent and independent of particle geometry (Figure 2d). Similar results were obtained when microparticles were normalized for silicon content (Figure S2).

Since all geometries of pSi microparticles promoted comparable secretion of the pro-inflammatory cytokine IL-1β, we investigated if these microparticles exhibited differing immunomodulatory effects *in vivo*. Female C57BL/6 mice were injected intraperitoneally (i.p.) with PBS or pSi microparticles in a range of geometries. The various pSi microparticle formulations induced comparable neutrophil (CD11b^+^ Gr1^high^ F4/80^−^; Figure 2e) and eosinophil (Gr1^−^ ckit^−^ SiglecF^+^; Figure 2f) recruitment into the peritoneum as well as a comparable reduction in the number of resident macrophages (CD11b^high^ F4/80^high^; Figure 2g) 24 hr after injection, indicating the ability of pSi microparticles to induce local inflammatory responses independent of geometry over the range of parameters used. The number of DCs (CD19^−^ CD11c^+^ MHCcII^+^), monocytes (CD19^−^ CD11c^−^ CD11b^+^ Ly6C^+^) and proinflammatory macrophages (CD19^−^ CD11c^−^ Gr1^low^ MHCcII^+^) were similar to PBS after injection with all geometries of microparticles (Figure S3).

Since D10.4 discoidal microparticles promoted secretion of the proinflammatory cytokine IL-1β, while exhibiting low levels of toxicity to BMDCs *in vitro*, we investigated the immunomodulatory effects of these particles in combination with MPL to evaluate potential synergistic induction of immune responses.

**Figure 3:**
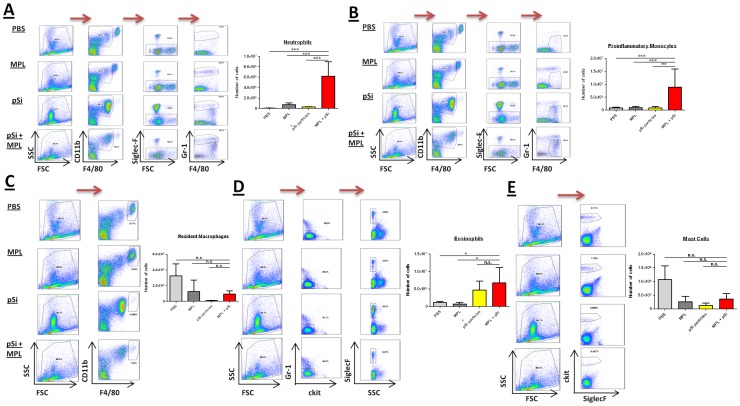
Injection of pSi microparticles with the TLR4 agonist MPL induces a synergistic recruitment of inflammatory cells. Female wild-type C57BL/6 mice were injected with PBS, MPL (50 µg/mouse), pSi microparticles (5×10^8^) or MPL plus pSi microparticles. The mice were sacrificed 24 hr later and PECs were isolated. PECs were stained with various markers to identify cell populations in the peritoneum at this time point, specifically the number of neutrophils (a; CD11b^+^ SiglecF^−^ Gr-1^+^), M1-like macrophages (b; CD11b^+^ SiglecF^−^ F4/80^+^ ), M2-like macrophages (c; CD11b^high^ F4/80^high^), eosinophils (d; Gr-1^−^ ckit^−^ SiglecF^−^) and mast cells (e; ckit^+^ SiglecF^−^). Indicated variable versus MPL+pSi, *p<0.05, ***p<0.001, n = 3/group.

Female C57BL/6 mice were injected i.p. with PBS, pSi microparticles, MPL or MPL adsorbed onto pSi microparticles and the mice were sacrificed after 24 hr. Injection of microparticles with MPL resulted in a pronounced synergistic effect on neutrophil (**Figure 3a**) and proinflammatory monocyte (CD11b^inter^ F4/80^inter^ Gr-1^low/inter^ SSC^low^; Figure 3b) recruitment into the peritoneum. While MPL alone did not drive the recruitment of inflammatory cells to the site of injection, pSi microparticles independently promoted eosinophil recruitment into the peritoneum (Figure 3d). Furthermore, the combination of MPL and pSi microparticles, as well as pSi microparticles alone, resulted in depletion of both resident macrophage (Figure 3c) and mast cell numbers (SiglecF^−^ ckit^+^; Figure 3e).

In order to determine whether pSi microparticles could modulate the cytokine profile of peritoneal exudate cells (PECs) at the injection site or in the draining lymph nodes, female C57BL/6 mice were injected i.p. with PBS, pSi microparticles, MPL or MPL absorbed onto pSi microparticles and the mice were sacrificed after 24 hr. PECs and mediastinal lymph node cells were isolated and restimulated *ex vivo* with the TLR9 agonist CpG or heat killed (HK) *E.coli*. Subsequently, secretion of the cytokines IFN-γ, TNF-α, IL-12p40 and IL-10 were measured.

**Figure 4:**
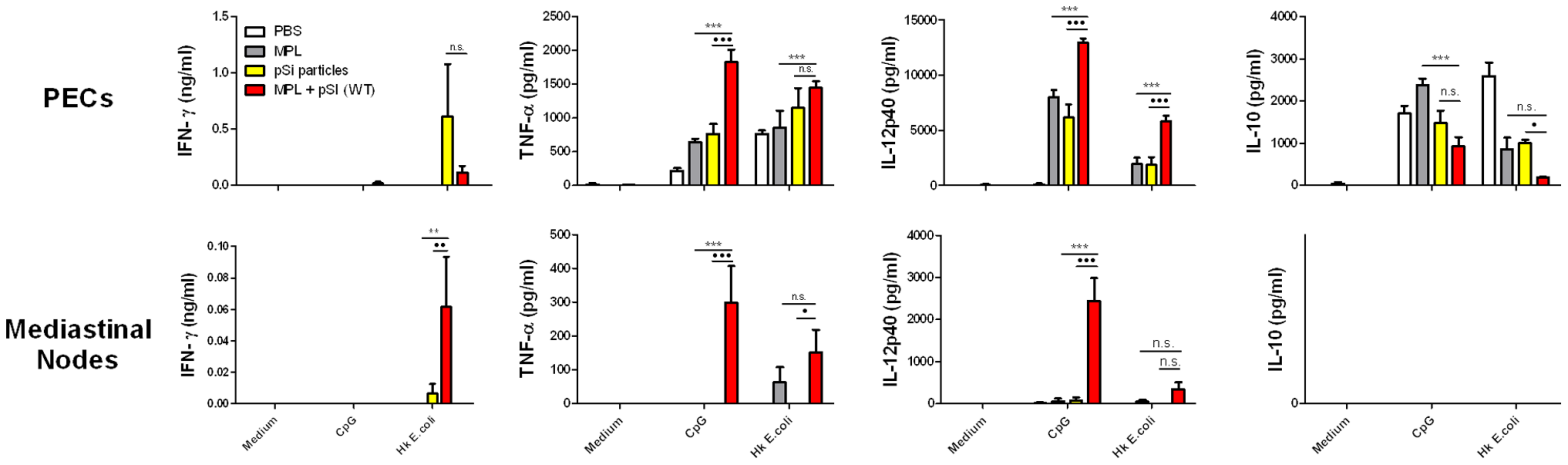
Injection of pSi microparticles with the TLR4 agonist MPL induces a synergistic production of proinflammatory cytokines. PECs were restimulated with CpG (5 µg/ml) or HK E. coli (10 E. coli : 1 PEC)(F). After 24 hr, supernatants were collected and analyzed for the cytokines IFN-γ, TNF-α, IL-12p40 and IL-10 by ELISA. MPL versus MPL-pSi, **p<0.01, ***p<0.001; pSi versus MPL-pSi, •p<0.05, •••p<0.001, n = 3/group.

PECs and mediastinal lymph node cells isolated from mice injected with MPL-pSi microparticles exhibited a significant increase in secretion of the proinflammatory cytokines IFN-γ, TNF-α and IL-12p40 (**Figure 4**). Injection of microparticles alone resulted in enhanced IFN-γ in response to stimulation with HK *E. coli*, as well as IL-12p40 and TNF-α in response to CpG or HK *E. coli*. In contrast, IL-10 production in response to both pathogen recognition receptor agonists was significantly reduced in these mice compared to controls. Injection of MPL alone was capable of priming PECs for enhanced IL-12p40 and TNF-α secretion in response to CpG. Production of these cytokines by PECs was significantly enhanced in mice injected with particles and MPL. In parallel, injection of MPL-pSi microparticles resulted in significantly less production of the anti-inflammatory cytokine IL-10 in comparison to cells from mice injected with either pSi microparticles or MPL alone. Importantly and in contrast to the PEC data, only the injection of MPL-pSi resulted in significantly enhanced IFN-γ, TNF-α and IL-12p40 production by restimulated mediastinal lymph node cells. Specifically lymph node cells from mice injected with MPL-pSi microparticles secreted TNF-α in response to both CpG and HK *E. coli*, as well as IL-12p40 in response to CpG and IFN-γ in response to HK *E. coli.*

### Suppression of Tumor Growth by MPL-pSi Microparticles and DOX-NPs

**Figure 5:**
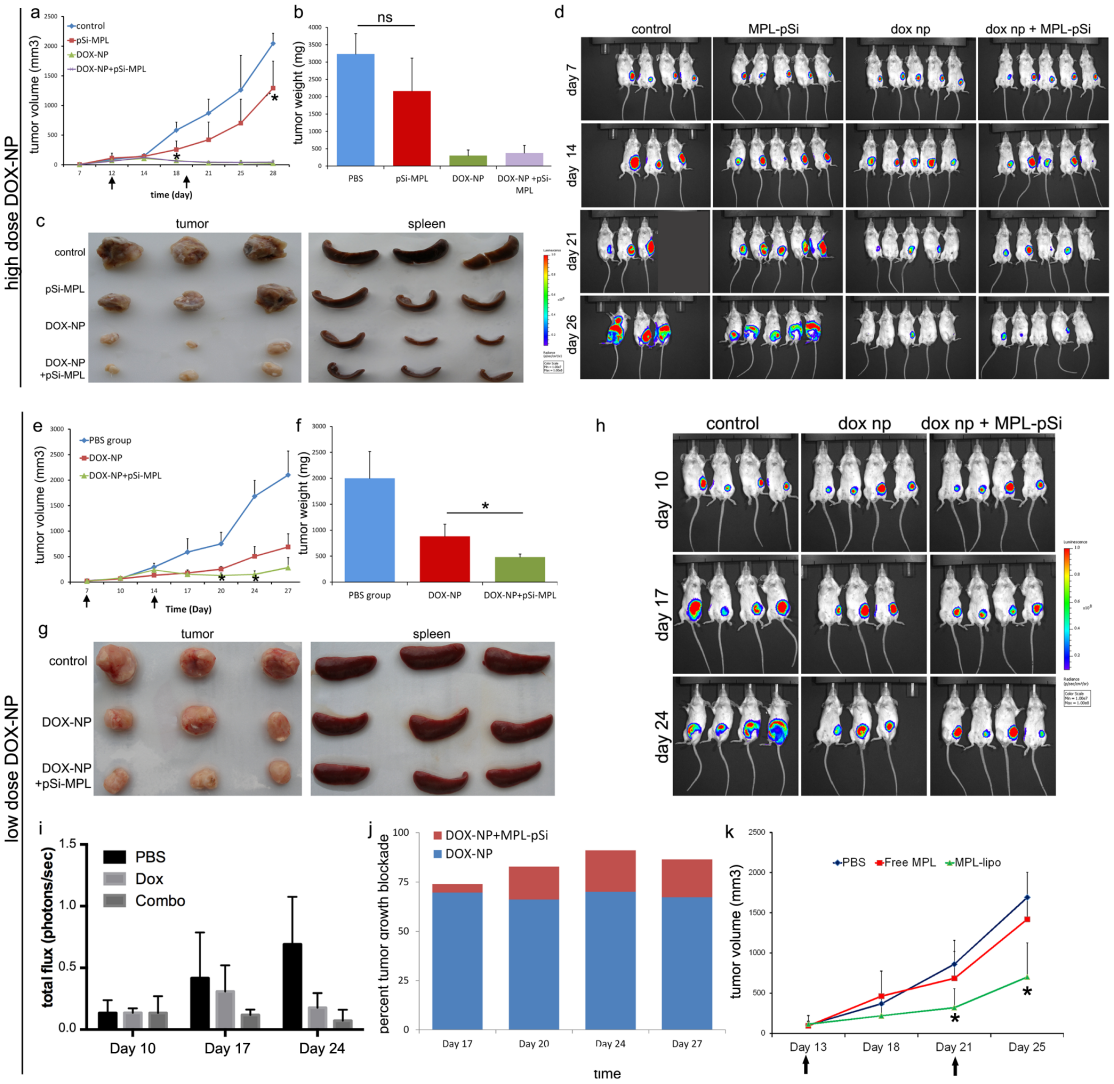
Combination therapy is superior to single agent therapy in a mouse model of breast cancer. a) 4T1 tumor growth in BALB/c mice was monitored using calipers. Mice were treated intravenously with MPL-pSi microparticles (5×10^8^) with and without high dose DOX-NPs weekly as indicated by arrows. Control verses MPL-pSi, *p<0.05, n = 3–5/group. b) Final mass of excised tumor. c) Images showing the relative size of extracted tumors and spleens. d) Mice were imaged weekly using the IVIS imaging system five min following injection with luciferin. e) Caliper-derived tumor volume measurements over time for BALB/c mice treated intravenously with low dose DOX-NPs in the presence or absence of MPL-pSi microparticles (5×10^8^) (time of microparticle injections indicated by arrows; DOX-NPs verses DOX-NPs plus MPL-pSi, *p<0.05, n = 3–4/group). f) Final weights of excised tumors, *p<0.05. g) Relative size of excised tumors and spleens. h) Weekly IVIS imaging of mice injected with luciferin. i) Quantitation of the bioluminescence data is presented as total flux in protons/sec. j) Increase in tumor growth blockade due to MPL-pSi microparticles (red) over that induced by DOX-NPs (blue) shown graphically across time. k) Similar to A & E, 4T1 tumor growth was monitored in BALB/c mice using calipers. Mice were injected with PBS (control), MPL-liposomes, or free MPL as indicated by the arrows (control vs MPL liposome, *p<0.05, n = 3/group).

To examine tumor growth inhibition by particle-presented chemotherapeutics and MPL, BALB/c mice bearing intramammary 4T1-luciferase breast tumors were injected with doxorubicin-loaded nanoliposomes (DOX-NPs; 5 mg/kg), MPL-pSi microparticles (5×10^8^; 10 µg MPL equivalent), or combined DOX-NPs and MPL-pSi by tail vein injection 7–12 days after tumor initiation. Tumor growth was monitored by caliper measurements and luciferase expression using the IVIS Imaging System 200. MPL-pSi microparticle treated mice exhibited significantly reduced tumor growth based on caliper measurements, and mice receiving combined MPL-pSi microparticles and DOX-NPs exhibited arrest of tumor growth (**Figure 5a**). The mean final tumor size is presented in Figure 5b, and actual tumor images are shown in Figure 5c. The majority of mice treated with MPL-pSi showed a visible reduction in tumor mass with no growth of tumors in mice treated with DOX-NPs. Inoculation of mice with MPL-pSi or DOX-NPs also reduced tumor-associated splenomegaly (Figure 5c). Images of photon emission from luciferase positive tumor cells are shown in Figure 5d, further supporting reduced tumor growth in the presence of MPL-pSi and showing complete loss of detectable tumor cells in most animals treated with DOX-NPs.

### Enhanced Tumor Ablation with Combined MPL-pSi and DOX-NPs Compared to Single Agent Therapy

While treatment with MPL-pSi reduced tumor growth when administered as a solo therapeutic agent, the synergistic or additive effect when co-administered with chemotherapy was masked due to strong anti-cancer effects at 5 mg/kg DOX-NPs. Therefore, mice were treated with half the effective DOX-NP dose, alone or in combination with MPL-pSi microparticles. Adjuvant MPL-pSi therapy significantly elevated suppression of tumor growth above that exhibited by treatment with DOX-NPs alone, as measured using calipers (Figure 5e) and based on the final tumor mass (Figure 5f). Visual inspection of the excised tumors and corresponding spleens (with reduced hypertrophy) are displayed in Figure 5g. Photon emission data from day 24 (Figure 5h) mirrored images of the excised tumors. Quantitation of the bioluminescence data is presented as total flux (photon/sec) in Figure 5i.

The relative increase in tumor blockade due to the addition of MPL microparticles to liposomal chemotherapy is displayed in Figure 5j. Blockade due to liposomes alone is shown in blue and the increase due to adjuvant microparticles is shown in red, supporting an additive effect of the combined therapy. We anticipate that the enhancing effect of microparticles will be even greater with smaller doses of DOX-NPs.

### Adjuvant Properties of MPL-nanoliposomes

To further determine if multivalent presentation of MPL is responsible for enhanced adjuvant properties compared to free MPL, we examined the impact of MPL-nanoliposomes and free MPL on 4T1 tumor growth. MPL-nanoliposomes had a mean size near 100 nm based on dynamic light scattering and the zeta potential was 40±2.5 mV. Similar to intravenous inoculation of mice with MPL-pSi microparticles, intratumoral inoculation with MPL-nanoliposomes reduced tumor growth significantly, while free MPL failed to alter tumor growth (Figure 5k).

### Alterations in Immune Cell Profiles of Tumor and Lymphatic Tissue Following Particle-based Therapy

**Figure 6:**
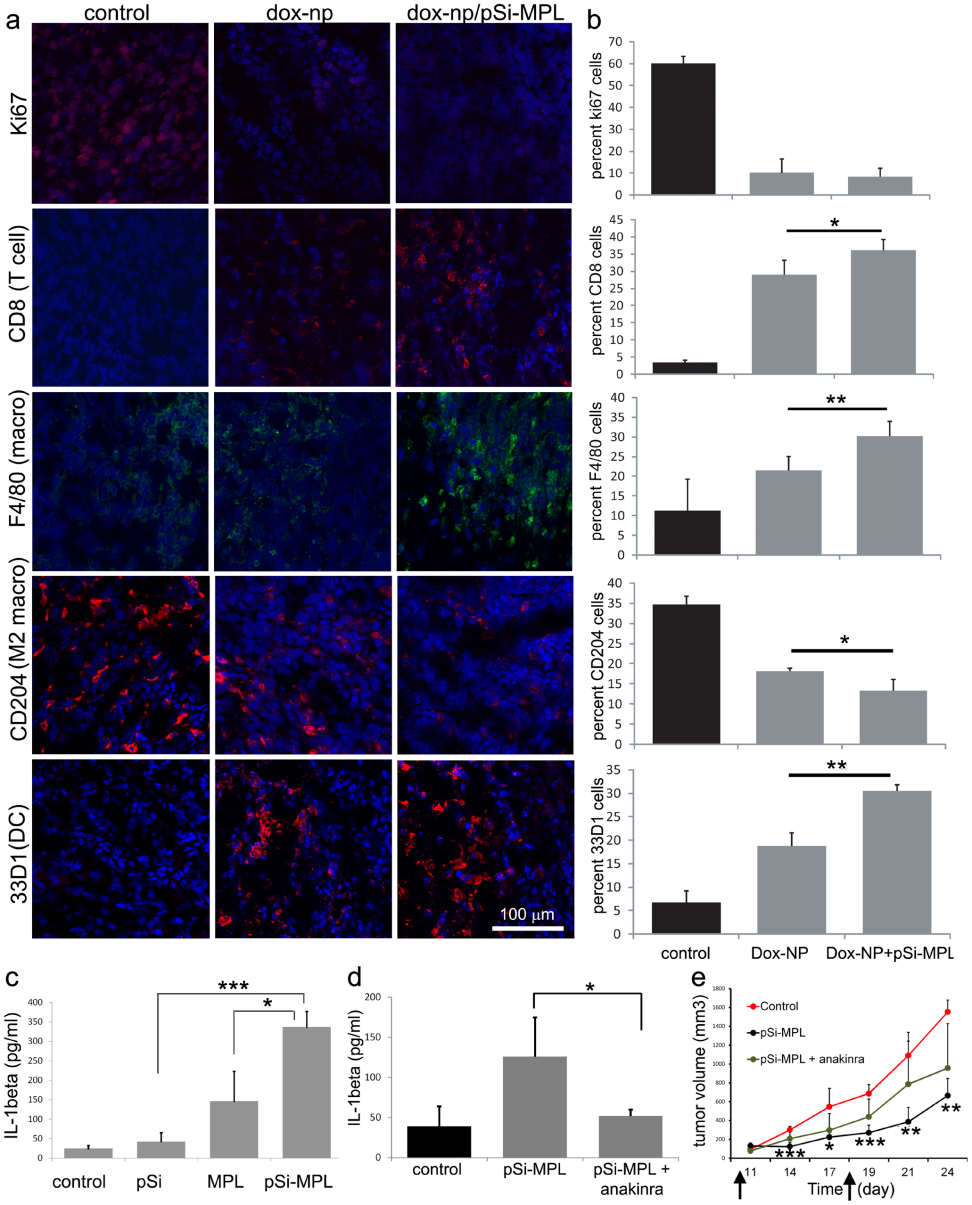
Combined chemo and MPL-pSi therapy inhibits cellular proliferation and stimulates tumor infiltration by immune cells which is partly IL-1 dependent. a) Excised tumors from mice treated with low dose DOX-NPs with or without pSi-MPL microparticles were stained with antibodies specific for Ki67 (red), CD8 (red), F4/80 (green), CD204 (red), or 33D1 (red). Nuclei were stained with Prolong Gold Antifade Reagent with DAPI (blue). b) The percentage of the population comprised of each cell type is shown graphically as the mean of 3–4 regions in randomly selected tissues representing at least two animals per group. *p<0.05; **p<0.01. c) Plasma IL-1β level six hr after intravenous injection of free MPL or 5×10^8^ control or MPL-pSi microparticles (10 µg MPL equivalents; n = 3/group; ***p<0.001; *p<0.05). d) Effect of anakinra on MPL-pSi microparticle-induced changes in plasma IL-1β six hr after injection (30 mg, 3×/week; *p<0.05; n = 3/group). e) Tumor growth of mice injected with MPL-pSi microparticles (weekly as indicated by arrows) with and without anakinra (30 mg, 3×/week; n = 3/group). Control verses MPL-pSi, *p<0.05; **p<0.01; ***p<0.001.

DOX-NP therapy caused a significant, dramatic reduction in the number of Ki67^+^ proliferating cells in the tumor (**Figure 6** **a,b**). In order to examine the impact of DOX-NPs (low dose), alone and in combination with MPL-pSi, on the population of tumor-infiltrating immune cells, we examined CD8^+^ cytotoxic T lymphocytes (CTL), F4/80^+^ and CD204^+^ myeloid cells, and 33D1^+^ DC in the tumors of control and particle-treated animals. Immunofluorescence staining of frozen tumor sections revealed increases in the proportion of both CTL and F4/80^+^ myeloid cells following DOX-NP treatment, with significant further increases induced by inoculation with MPL-pSi (Figure 6a,b). 33D1^+^ DC were similarly increased; however, the majority of DCs were localized to the tumor periphery. Conversely, CD204^+^ alternatively activated M2 macrophages were reduced in the tumors of particle-treated mice.

Mice administered MPL-pSi intravenously displayed significantly elevated IL-1β in the serum six hr after treatment compared to mice treated with control pSi microparticles (*p<0.0002) or free MPL [(**p<0.02; Figure 6c]. Anakinra (Kineret), a recombinant soluble IL-1 receptor antagonist, blocked IL-1β secretion in MPL-pSi treated mice (Figure 6d) and reduced the anti-tumor efficacy of the microparticles, however, due the small number of mice used in the study, tumor size in these mice was not significantly different from that of control animals (n = 3/group; Figure 6e).

Myeloid populations were also examined by immunofluorescence staining of the draining lymph nodes and spleen of particle-treated mice. For mice treated with a therapeutic dose of DOX-NPs, F4/80^+^ and CD204^+^ myeloid cells in the draining lymph nodes were phenotypically similar to control and combination treated mice nine days following the final injection of DOX-NPs and pSi-MPL (Figure S4 a,b; middle row). Similar data was obtained for the spleen with respect to F4/80^+^ and CD204^+^ macrophages (Figure S4 a,b; top row). The population of Gr-1^+^/CD11b^+^ myeloid derived suppressor cells (MDSC) in the spleen were similar with respect to percentage of MDSC, however, since the size of the spleens was drastically reduced in treated mice, the absolute number of MDSC was correspondingly reduced with treatment. Interestingly, the percentage of Gr-1^+^/CD11b^−^ cells increased in mice treated with combination DOX-NP and MPL-pSi microparticles.

## Discussion

Following internalization of tumor antigens, mature DCs are able to activate T lymphocytes having high tumor antigen avidity, leading to antigen-specific cytolytic responses [25]. Herein we show that pSi microparticles are internalized by DC, regardless of their geometric presentation within the study parameters, and in contrast to alum, are nontoxic. Furthermore, pSi microparticles stimulate NLRP3 inflammasome dependent IL-1β secretion by DCs.

IL-12 has potent anti-tumor effects, based in part on blockade of T regulatory cell secretion of IL-10 and TGF-β. [26] Since alum can suppress IL-12 secretion by DCs [4] and is not an effective promoter of Th1 responses, alternative adjuvant systems for cancer vaccines are required. In this study, in situ multivalent presentation of MPL by pSi microparticles elevated MPL-induced secretion of the Th1 cytokines IFN-γ, and TNF-α, and decreased levels of the immunosuppressive cytokine IL-10. Injection of MPL-pSi also favored the recruitment of neutrophils, eosinophils and proinflammatory monocytes, while reducing numbers of resident macrophages and mast cells.

As cancer progresses, tumor cells evade immune defenses and become increasingly refractory to chemotherapies. Tumors are rich in MDSC, a heterogeneous cell population that has characteristics of both M1 and M2 macrophages and inhibits both innate and adaptive immunity. [27] Since a variety of chemotherapeutics, including gemcitabine and doxorubicin, are reported to decrease MDSCs and thereby have favorable immunologic effects, leading to reduced splenomegaly in addition to blockade of tumor growth, [28] we evaluated the population of MDSC in the spleen of control and treated mice. While there was no significant difference between control and treated mice with respect to percentage of Gr-1^+^/CD11b^+^ cells on day 9 after the second injection with particles, the absolute number of MDSC was reduced to a degree corresponding to the large reduction in spleen mass. Similar results were reported by Alizadeh et al. [29], who observed lower percentages of MDSC on days 2 and 5 after doxorubicin treatment of mice with 4T1 tumors, but no difference of day 11. We did however observe an increase in the percentage of Gr-1^+^ cells in the spleen of mice receiving combination therapy, which may represent Gr-1^+^/CD11b^−^ plasmacytoid DCs [30].

This study demonstrated that injection of mice bearing 4T1 tumors with MPL-pSi microparticles independently reduced tumor growth and stimulated a Th1 bias in the tumor microenvironment. Mice receiving DOX-NPs exhibited increases in the percentage of CTLs, F4/80^+^ macrophages, and DC in the tumor, with further increases induced by inoculation with MPL-pSi. Conversely, injection of DOX-NPs decreased the proportion of CD204^+^ macrophages, which are associated with tumor aggressiveness, [31] with augmentation of the effect stimulated by addition of MPL-pSi. Anakinra, a competitive inhibitor of IL-1, blocked MPL-pSi induced secretion of IL-1β, and reduced the anti-tumor properties of the microparticles. In summary, multivalent presentation of MPL by pSi enables a bias towards Th1 polarization, functioning as an attractive adjuvant for combination immunotherapy and for future applications in vaccine development.

## Supporting Information

Figure S1 **Influence of alum and pSi microparticles on cell viability.** D10.4, D25.4 or R18.4.4 pSi microparticles were normalized for silicon content and added to LPS primed (10 ng/ml) BMDCs at a top concentration of 0.006 mg/ml silicon, parallel to alum (50 µg/ml) or LPS (10 ng/ml) for 24 hr. Percent cell death, based on propidium iodide uptake, is displayed.
(DOCX)

Figure S2 **pSi microparticle-stimulated secretion of IL-1β.** Microparticles were normalized for silicon content and added to LPS primed (10 ng/ml) WT (top) and NLRP3^−/−^ (bottom) BMDCs in decending concentrations with a top concentration of 0.006 mg/ml Si. IL-1β secretion was determined by ELISA.
(DOCX)

Figure S3 **Influence of alum and pSi microparticle geometry on number of DC, monocytes and pro-inflammatory macrophages recruited to site of injection.** Female wild-type C57BL/6 mice were injected intra-peritoneally with PBS, Alum (0.3 mg/mouse) or pSi microparticles with geometries D10.2, D10.4, D25.4 or RS18.4.4 (0.3 mg/mouse). The mice were sacrificed 24 hr later and PECs were isolated and stained for flow cytometry to determine numbers of DC (a), monocytes (b) and pro-inflammatory macrophages (c).
(DOCX)

Figure S4 **Macrophage and MDSC populations in the draining lymph node and spleen of treated mice.** a) Spleen and lymph node sections from BALB/c mice treated with DOX-NPs and MPL pSi microparticles were stained with fluorescent antibodies for F/80 (green) and CD204 (red) macrophages; and Gr-1 (green) and CD11b (red) MDSCs. b) The proportion of positive cells in each population was determined in randomly selected tissues selecting 4–6 regions of interest based on DAPI staining and then counting cell populations using Image J software and the ITCN plugin. The percentage of positive cells in each population was determined by dividing the number of cells of interest by the total number of cells based on DAPI staining.
(DOCX)
