# Correction: Multivalent Presentation of MPL by Porous Silicon Microparticles Favors T Helper 1 Polarization Enhancing the Anti-Tumor Efficacy of Doxorubicin Nanoliposomes

**DOI:** 10.1371/journal.pone.0314577

**Published:** 2024-11-21

**Authors:** Ismail M. Meraz, Claire H. Hearnden, Xuewu Liu, Marie Yang, Laura Williams, David J. Savage, Jianhua Gu, Jessica R. Rhudy, Kenji Yokoi, Ed C. Lavelle, Rita E. Serda

Two panels in Fig 5 of this article [1] are incorrect: the Fig 5D Day 7 Control panel and the Fig 5H Day 10 dox np panel. These errors are corrected in the updated version of Fig 5 provided here. The underlying image data for Fig 5 are provided with this notice in S1 File, and the data for other figures are available upon request from the corresponding author.

The Materials and Methods section of [1] does not include specific information about humane endpoints used in the study. Humane endpoints included a tumor size equal or greater to 2.0 cm in any direction, loss of body weight of 20%, low activity, ruffled fur, hunched back, respiratory distress, or other signs indicating a body condition score equal or less than 2. The corresponding author stated, “our research team (researchers) checked the animals on a daily basis, especially toward the later days of the experiment. Early on we were checking 2–3 days/week. The vivarium did have a vet or vet tech who checked animal health daily and if they identified a health concern, they would email the research fellows. Mice were evaluated for tumor size (calipers) and signs of distress (coat appearance, activity level, weight loss, shaking or posture changes). Some animals were euthanized early. Animals were euthanized early if the animal’s health declined significantly and/or if the veterinarian recommended sacrificing. This was not reported to an ethics board because we were following the IACUC approved protocol at Houston Methodist Research Institute. There was no variation from our protocol to warrant reporting to the ethics committee.”

**Fig 5:**
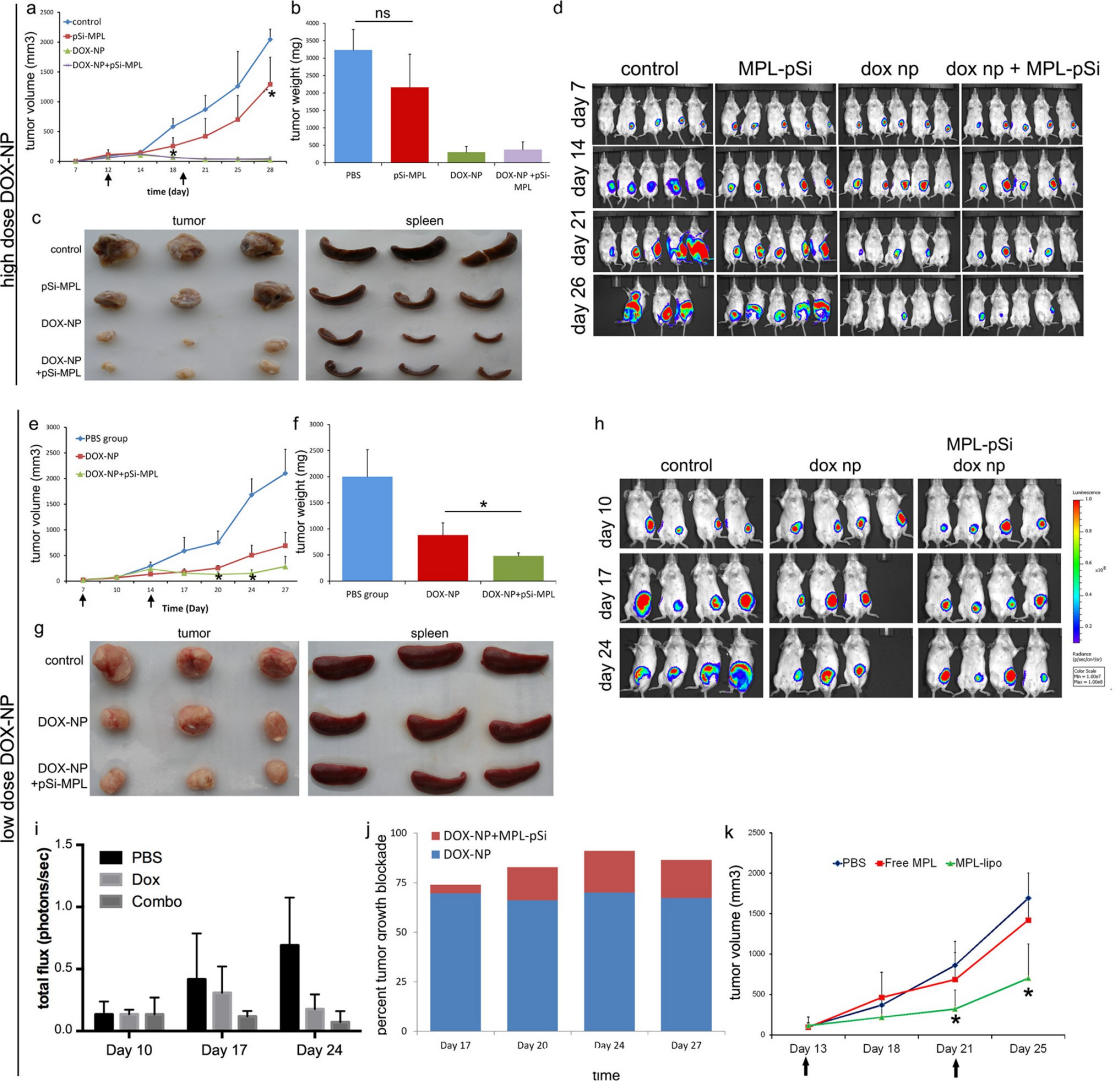
Combination therapy is superior to single agent therapy in a mouse model of breast cancer. a) 4T1 tumor growth in BALB/c mice was monitored using calipers. Mice were treated intravenously with MPL-pSi microparticles (5×10^8^) with and without high dose DOX-NPs weekly as indicated by arrows. Control verses MPL-pSi, *p<0.05, n  =  3–5/group. b) Final mass of excised tumor. c) Images showing the relative size of extracted tumors and spleens. d) Mice were imaged weekly using the IVIS imaging system five min following injection with luciferin. e) Caliper-derived tumor volume measurements over time for BALB/c mice treated intravenously with low dose DOX-NPs in the presence or absence of MPL-pSi microparticles (5×10^8^) (time of microparticle injections indicated by arrows; DOX-NPs verses DOX-NPs plus MPL-pSi, *p<0.05, n  =  3–4/group). f) Final weights of excised tumors, *p<0.05. g) Relative size of excised tumors and spleens. h) Weekly IVIS imaging of mice injected with luciferin. i) Quantitation of the bioluminescence data is presented as total flux in protons/sec. j) Increase in tumor growth blockade due to MPL-pSi microparticles (red) over that induced by DOX-NPs (blue) shown graphically across time. k) Similar to A & E, 4T1 tumor growth was monitored in BALB/c mice using calipers. Mice were injected with PBS (control), MPL-liposomes, or free MPL as indicated by the arrows (control vs MPL liposome, *p<0.05, n  =  3/group).

## Supporting information

S1 File Raw image data to support Fig 5. (ZIP)
