# Supplementary material for: Multivalent Presentation of MPL by Porous Silicon Microparticles Favors T Helper 1 Polarization Enhancing the Anti-Tumor Efficacy of Doxorubicin Nanoliposomes
Source: PLoS One. 2014 Apr 15;9(4):e94703. doi: 10.1371/journal.pone.0094703 (PMC3988134; doi:10.1371/journal.pone.0094703)
Supplement: Figure S1 — Influence of alum and pSi microparticles on cell viability. D10.4, D25.4 or R18.4.4 pSi microparticles were normalized for silicon content and added to LPS primed (10 ng/ml) BMDCs at a top concentration of 0.006 mg/ml silicon, parallel to alum (50 µg/ml) or LPS (10 ng/ml) for 24 hr. Percent cell death, based on propidium iodide uptake, is displayed. (DOCX) [file pone.0094703.s001.docx]

Cytotoxicity of adjuvants: Measurement of BMDC necrosis by propidium iodine staining

Microparticle compatibility with primary BMDC was assessed using flow cytometry to measure cell viability. Microparticles were normalized by silicon content and added to LPS primed (10 ng/ml) wildtype (WT) BMDCs (6.25 x 10^5^ cells/ml) at a top concentration of 0.006 mg/ml silicon. BMDCs were incubated with alum (50 µg/ml), LPS (10 ng/ml), or D10.4, D25.4 or R18.4.4 pSi microparticles for 24 hr. Cells were centrifuged and resuspended in PBS. Cellular necrosis was evaluated based on propidium iodide (1 µg/ml) uptake, added just before acquisition. Samples were acquired using summit software (Dako, Colorado, USA) and the data were analysed using FlowJo^TM^ software (Treestar, Oregon, USA). When microparticle toxicity was compared using equivalent doses of silicon across the spectrum, the largest particles (D25.4) displayed low levels of toxicity at the highest dose (Supplemental Figure 1).


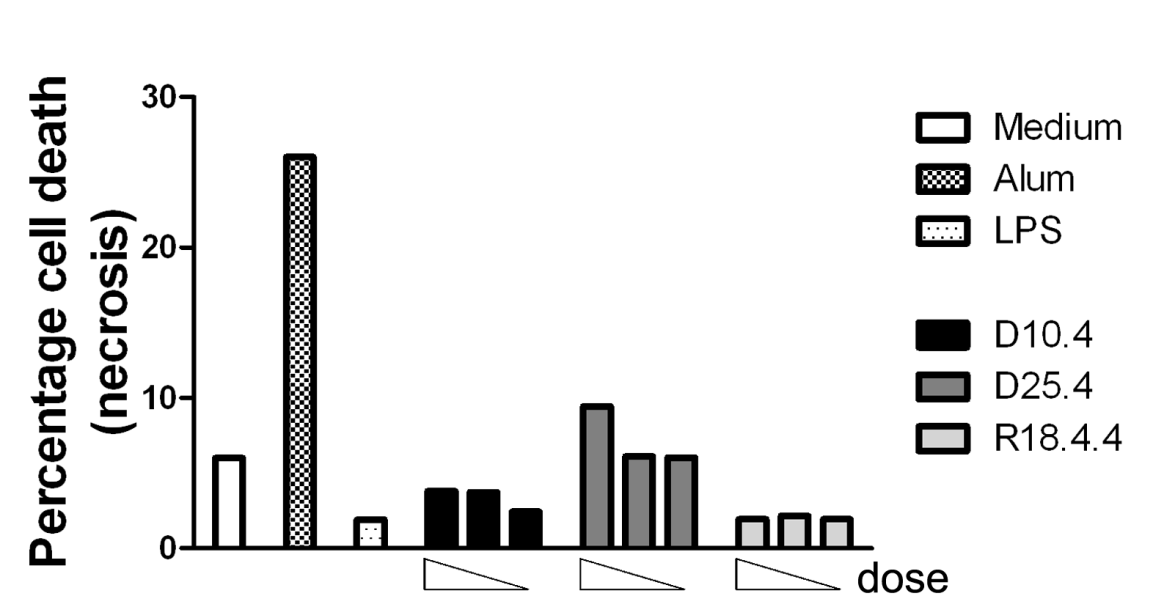


**Figure S1. Influence of alum and pSi microparticles on cell viability.** D10.4, D25.4 or R18.4.4 pSi microparticles were normalized for silicon content and added to LPS primed (10 ng/ml) BMDCs at a top concentration of 0.006 mg/ml silicon, parallel to alum (50 µg/ml) or LPS (10 ng/ml) for 24 hr. Percent cell death, based on propidium iodide uptake, is displayed.
