# Supplementary material for: Multivalent Presentation of MPL by Porous Silicon Microparticles Favors T Helper 1 Polarization Enhancing the Anti-Tumor Efficacy of Doxorubicin Nanoliposomes
Source: PLoS One. 2014 Apr 15;9(4):e94703. doi: 10.1371/journal.pone.0094703 (PMC3988134; doi:10.1371/journal.pone.0094703)
Supplement: Figure S2 — pSi microparticle-stimulated secretion of IL-1β. Microparticles were normalized for silicon content and added to LPS primed (10 ng/ml) WT (top) and NLRP3−/− (bottom) BMDCs in decending concentrations with a top concentration of 0.006 mg/ml Si. IL-1β secretion was determined by ELISA. (DOCX) [file pone.0094703.s002.docx]

Influence of pSi microparticles on IL-1β secretion from normal and NLRP3^-/-^ BMDC

The ability to pSi microparticles to stimulate IL-1β secretion from normal and NLRP3^-/-^ BMDC was tested using BMDC, plated at 6.25 x 10^5^ cells/ml, and primed with LPS at 1 ng/ml or 10 ng/ml for 1 hr before silicon microparticles were added. The dose of pSi microparticles was normalized for silicon content. Alum was used at 50 µg/ml or polydAdt at 5 µg/ml. After 24 hr, supernatants were collected and IL-1β concentrations were determined by ELISA. LPS-induced secretion of the pro-inflammatory cytokine IL-1β by BMDC was elevated by the presence of pSi microparticles, with larger microparticles inducing greater levels of stimulation at equivalent mass doses (Supplemental Figure 2). Secretion of IL-1β secretion, with the exception of polydAdt, was completely blocked in LPS treated NLRP3^-/-^ mice.


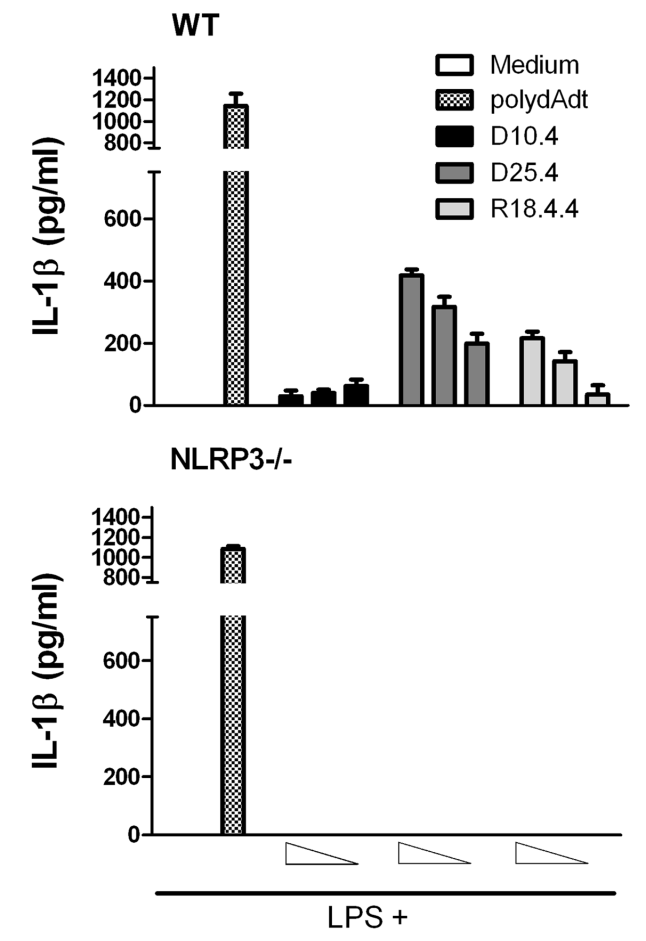


**Figure S2. pSi microparticle-stimulated secretion of IL-1β**. Microparticles were normalized for silicon content and added to LPS primed (10 ng/ml) WT (top) and NLRP3^-/-^ (bottom) BMDCs in decending concentrations with a top concentration of 0.006 mg/ml Si. IL-1β secretion was determined by ELISA.
