# Supplementary material for: Multivalent Presentation of MPL by Porous Silicon Microparticles Favors T Helper 1 Polarization Enhancing the Anti-Tumor Efficacy of Doxorubicin Nanoliposomes
Source: PLoS One. 2014 Apr 15;9(4):e94703. doi: 10.1371/journal.pone.0094703 (PMC3988134; doi:10.1371/journal.pone.0094703)
Supplement: Figure S3 — Influence of alum and pSi microparticle geometry on number of DC, monocytes and pro-inflammatory macrophages recruited to site of injection. Female wild-type C57BL/6 mice were injected intra-peritoneally with PBS, Alum (0.3 mg/mouse) or pSi microparticles with geometries D10.2, D10.4, D25.4 or RS18.4.4 (0.3 mg/mouse). The mice were sacrificed 24 hr later and PECs were isolated and stained for flow cytometry to determine numbers of DC (a), monocytes (b) and pro-inflammatory macrophages (c). (DOCX) [file pone.0094703.s003.docx]

Intra-peritoneal injection of pSi microparticles does not induce the recruitment of dendritic cells, monocytes or pro-inflammatory macrophages, irrespective of pSi microparticle geometry.

The ability of the different geometries of pSi microparticles to induce the recruitment of specific innate cells to the site of injection was determined. Specifically, dendritic cells (CD19^-^ CD11c^+^ MHCcII^+^), monocytes (CD19^-^ CD11c^-^ CD11b^+^ Ly6C^+^) and pro-inflammatory macrophages (CD19^-^ CD11c^-^ Gr1^low^ MHCcII^+^) were gated as described and the total number of cells in each population was determined. Injection with alum induced the recruitment of monocytes and pro-inflammatory macrophages, however mice injected with pSi microparticles had similar numbers of dendritic cells (a), monocytes (b) and pro-inflammatory macrophages (c) to those injected with PBS (Supplemental Figure 3).

**
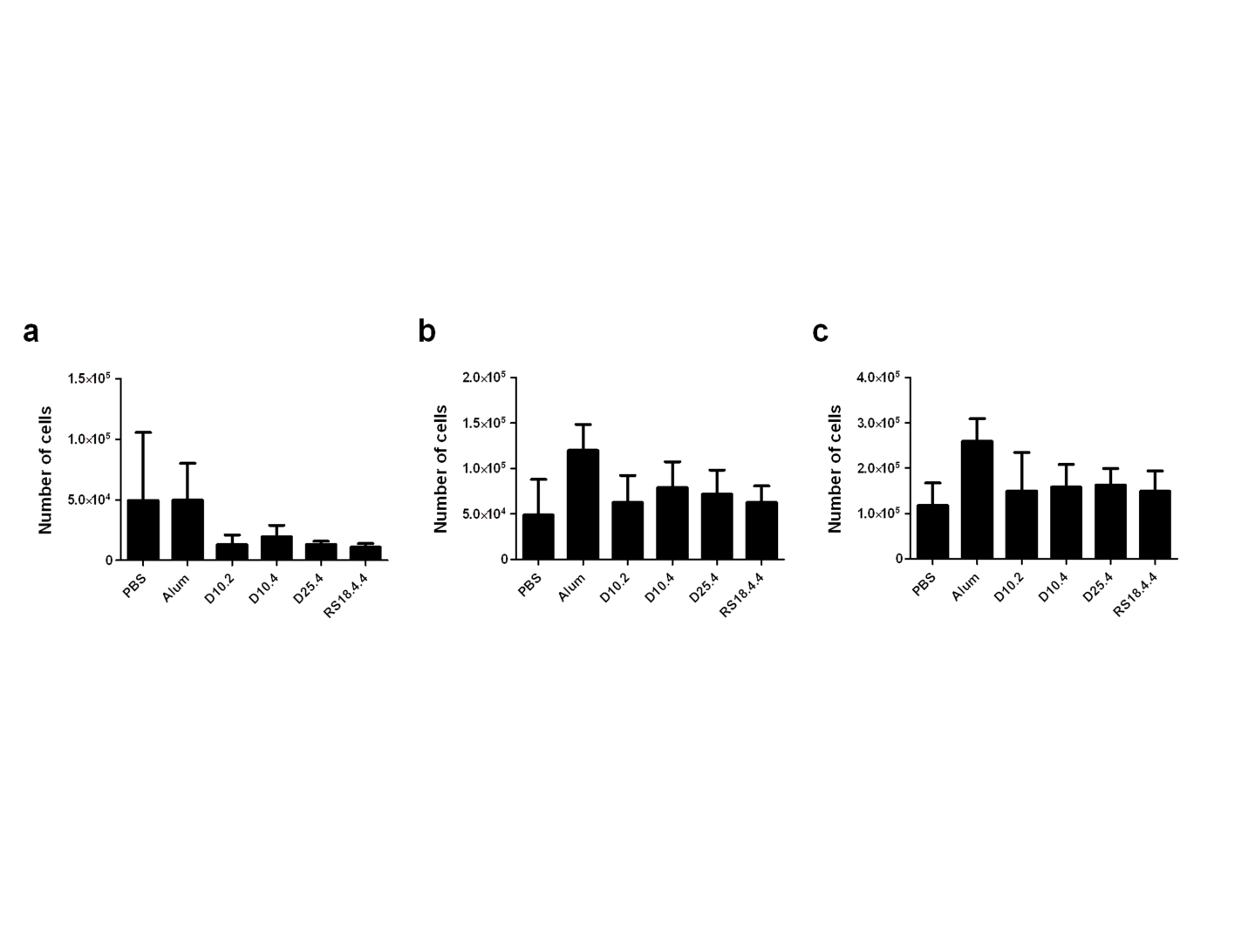
**

**Figure S3. Influence of alum and pSi microparticle geometry on number of dendritic cells, monocytes and pro-inflammatory macrophages recruited to site of injection**. Female wild-type C57BL/6 mice were injected intra-peritoneally with PBS, Alum (0.3 mg/mouse) or pSi microparticles with geometries D10.2, D10.4, D25.4 or RS18.4.4 (0.3 mg/mouse). The mice were sacrificed 24 hr later and PECs were isolated and stained for flow cytometry to determine numbers of dendritic cells (a), monocytes (b) and pro-inflammatory macrophages (c).
