# Supplementary material for: Multivalent Presentation of MPL by Porous Silicon Microparticles Favors T Helper 1 Polarization Enhancing the Anti-Tumor Efficacy of Doxorubicin Nanoliposomes
Source: PLoS One. 2014 Apr 15;9(4):e94703. doi: 10.1371/journal.pone.0094703 (PMC3988134; doi:10.1371/journal.pone.0094703)
Supplement: Figure S4 — Macrophage and MDSC populations in the draining lymph node and spleen of treated mice. a) Spleen and lymph node sections from BALB/c mice treated with DOX-NPs and MPL pSi microparticles were stained with fluorescent antibodies for F/80 (green) and CD204 (red) macrophages; and Gr-1 (green) and CD11b (red) MDSCs. b) The proportion of positive cells in each population was determined in randomly selected tissues selecting 4–6 regions of interest based on DAPI staining and then counting cell populations using Image J software and the ITCN plugin. The percentage of positive cells in each population was determined by dividing the number of cells of interest by the total number of cells based on DAPI staining. (DOCX) [file pone.0094703.s004.docx]

DOX-NPs and adjuvant microparticles do not alter the proportion of F4/80 and CD204 macrophages in the draining lymph node

Changes in the composition of macrophages in the draining lymph nodes of mice treated with therapeutic doses of DOX-NPs and microparticles were evaluated by immunofluorescence staining of tissue sections using F4/80 and CD204 antibodies. On the day of sacrifice, 9 days following the final dose of DOX-NPs and pSi-MPL, the selected macrophage populations were counted using Image J software (National Institute of Health) and the ITCN (Image-based tool for counting nuclei) Plugin (Thomas Kuo and Jiyun Byun; Center for Bio-image Informatics at UC Santa Barbara, CA, USA). The proportion of F4/80^+^ and CD204^+^ macrophages in the draining lymph nodes were phenotypically similar for control, DOX-NP, and combination DOX-NP plus pSi-MPL treated mice (Figure S4 a,b; middle row). Similar results were obtained for the spleen with respect to F4/80^+^ and CD204^+^ macrophages (Figure S4 a,b; top row).

DOX-NP and adjuvant microparticle therapy reduces the number but not the proportion of MDSC in the spleen.

The population of Gr-1^+^/CD11b^+^ MDSCs in the spleen were similar with respect to percentage of cells, however, since the size of the spleens was drastically reduced in treated mice, the absolute number of MDSC was correspondingly reduced. Interestingly, the percentage of Gr-1^+^/CD11b^-^ cells increased in mice treated with DOX-NPs plus MPL-pSi microparticles compared to control or DOX-NP groups (Figure S4 a,b; bottom row).


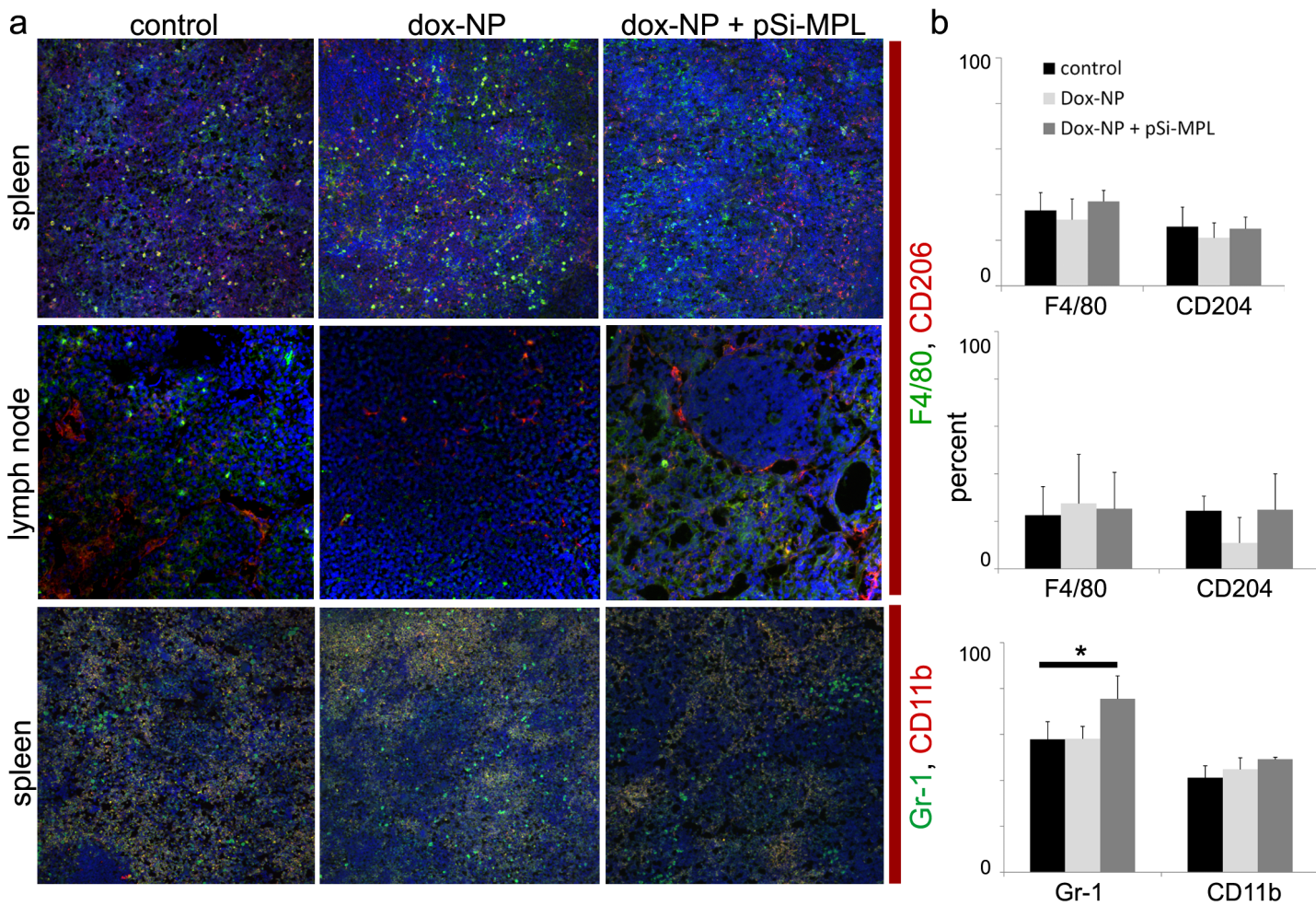


**Figure S4. Macrophage and MDSC populations in the draining lymph node and spleen of treated mice.** a) Spleen and lymph node sections from BALB/c mice treated with DOX-NPs and MPL pSi microparticles were stained with fluorescent antibodies for F4/80 (green) and CD204 (red) macrophages; and Gr-1 (green) and CD11b (red) MDSCs. b) The proportion of positive cells in each population was determined in randomly selected tissues selecting 4-6 regions of interest based on DAPI staining and then counting cell populations using Image J software and the ITCN plugin. The percentage of positive cells in each population was determined by dividing the number of cells of interest by the total number of cells based on DAPI staining.
