# Supplementary figures and images for: Correction: Multivalent Presentation of MPL by Porous Silicon Microparticles Favors T Helper 1 Polarization Enhancing the Anti-Tumor Efficacy of Doxorubicin Nanoliposomes
Source: PLoS One. 2024 Nov 21;19(11):e0314577. doi: 10.1371/journal.pone.0314577 (PMC11581198; doi:10.1371/journal.pone.0314577)

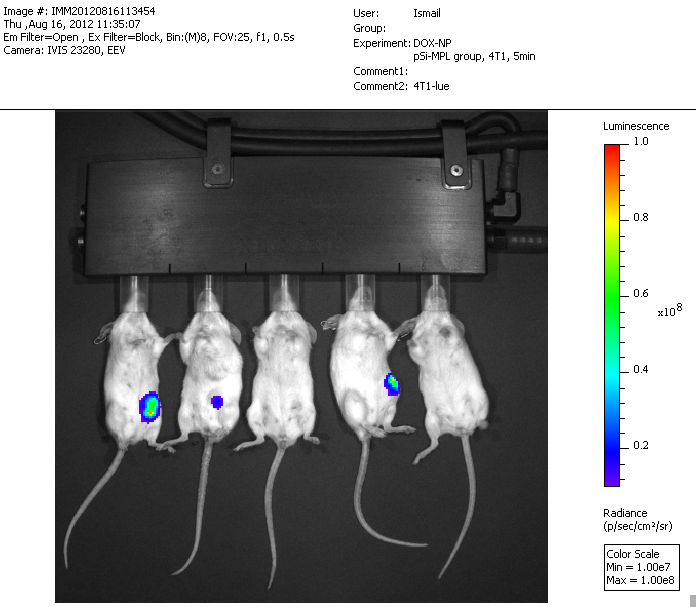

Supplement: S1 File — (ZIP) [file pone.0314577.s001.zip › S1 File/Fig 5D/26 DOX-NP + pSi-MPL.tif]

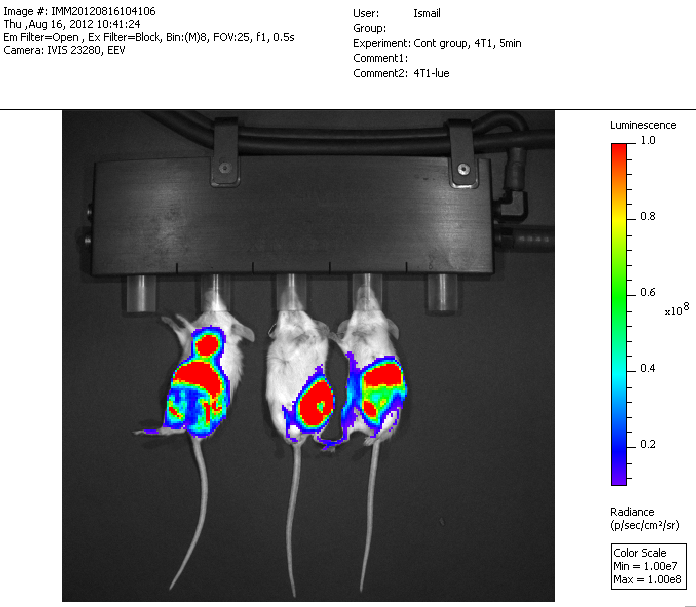

Supplement: S1 File — (ZIP) [file pone.0314577.s001.zip › S1 File/Fig 5D/26 PBS.tif]

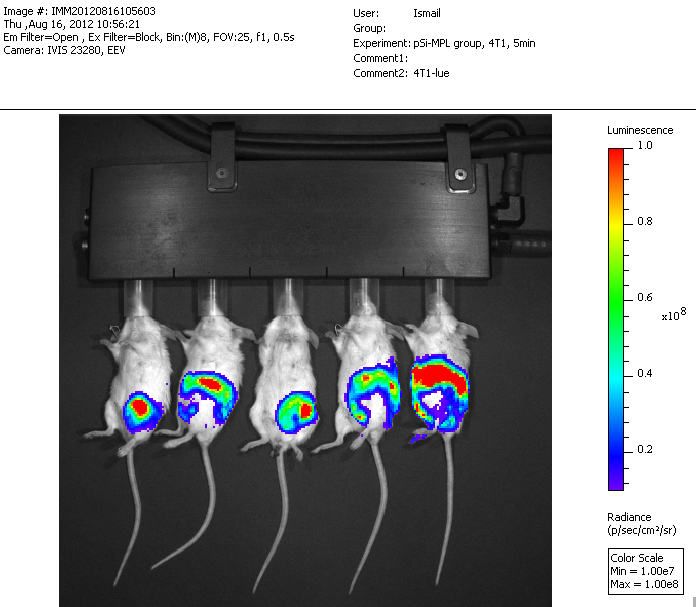

Supplement: S1 File — (ZIP) [file pone.0314577.s001.zip › S1 File/Fig 5D/26 pSi-MPL.tif]

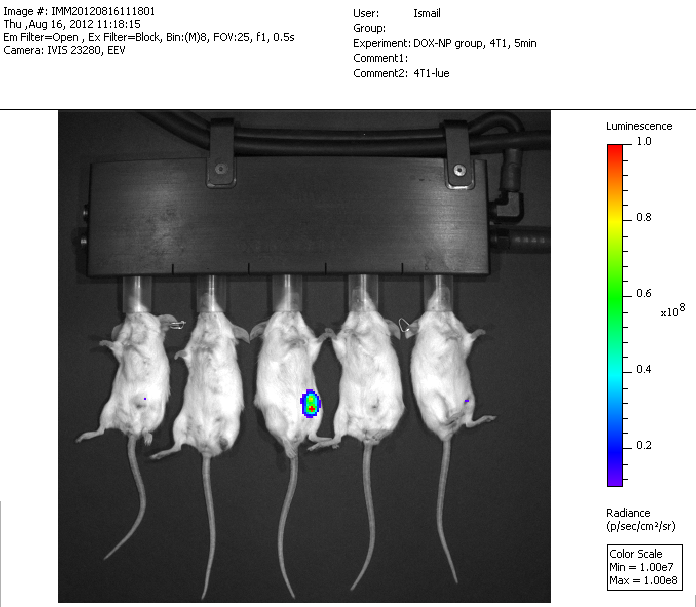

Supplement: S1 File — (ZIP) [file pone.0314577.s001.zip › S1 File/Fig 5D/26 DOX-NP.tif]

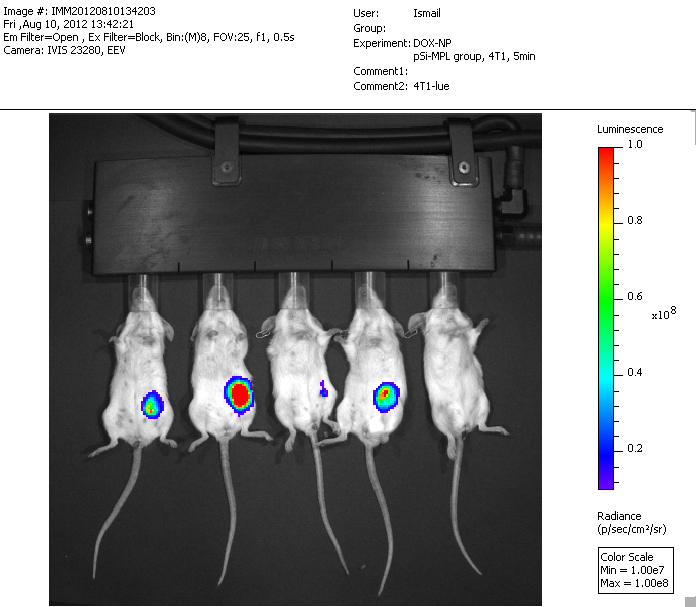

Supplement: S1 File — (ZIP) [file pone.0314577.s001.zip › S1 File/Fig 5D/21 Dox-NP+pSi-MPL.tif]

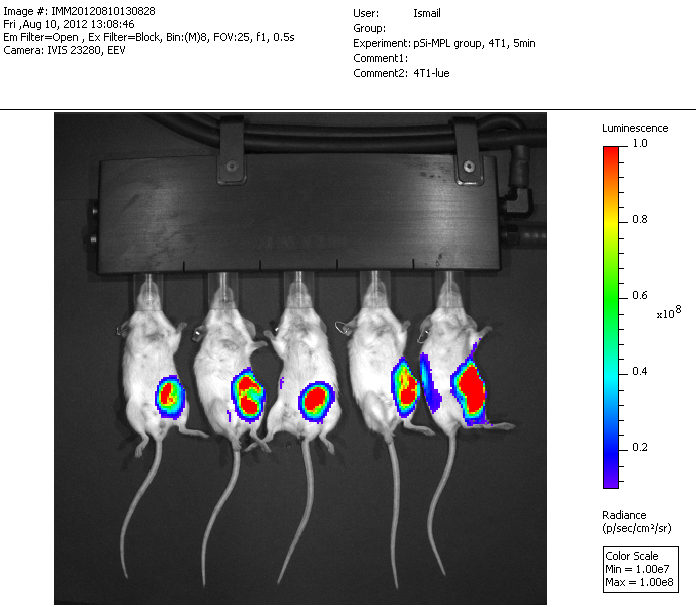

Supplement: S1 File — (ZIP) [file pone.0314577.s001.zip › S1 File/Fig 5D/21 pSi-MPL.tif]

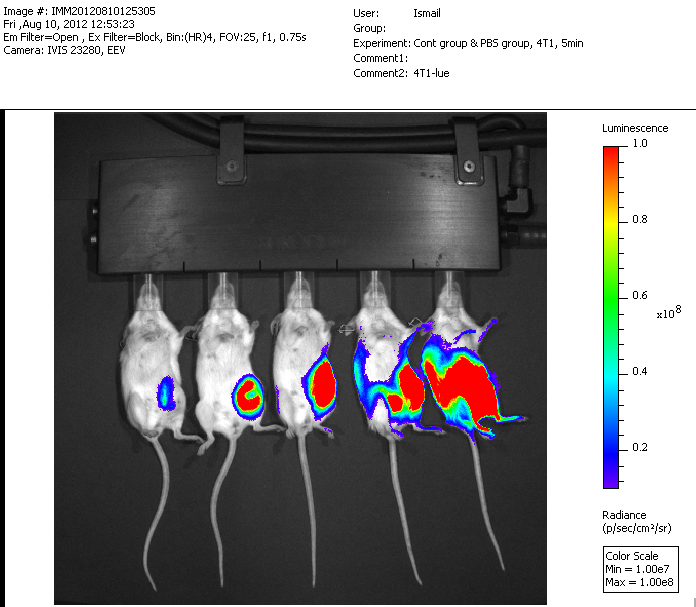

Supplement: S1 File — (ZIP) [file pone.0314577.s001.zip › S1 File/Fig 5D/21 PBS.tif]

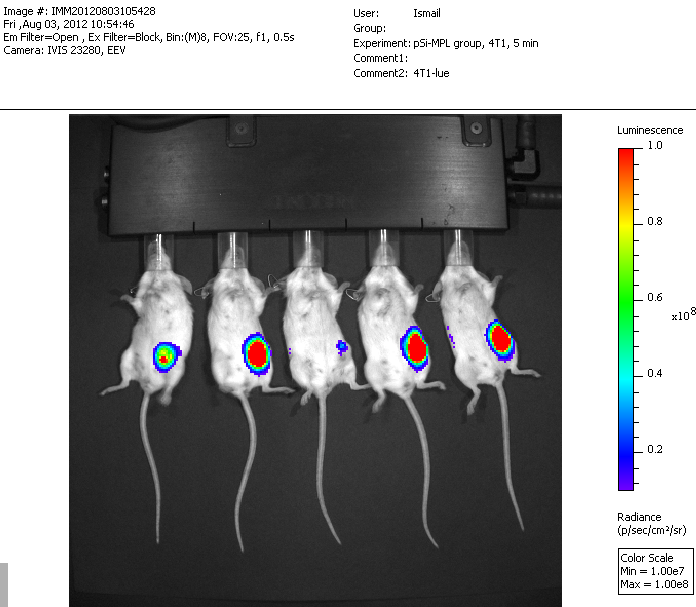

Supplement: S1 File — (ZIP) [file pone.0314577.s001.zip › S1 File/Fig 5D/14 pSi-MPL.tif]

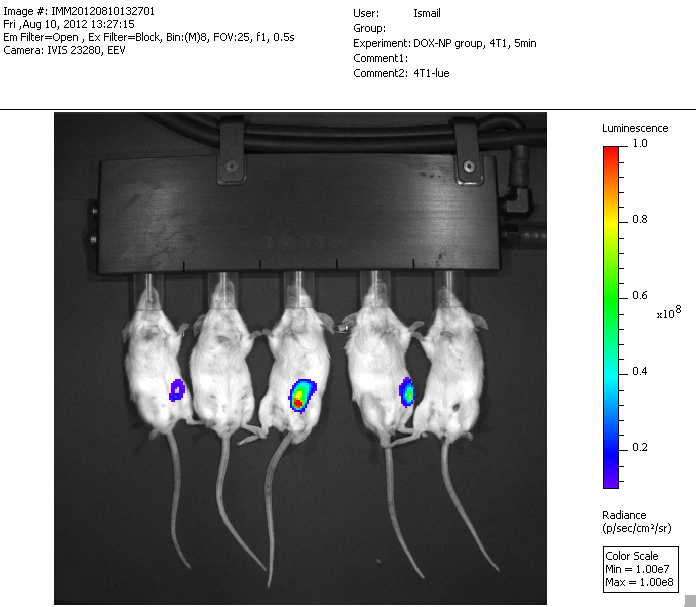

Supplement: S1 File — (ZIP) [file pone.0314577.s001.zip › S1 File/Fig 5D/21 Dox-NP.tif]

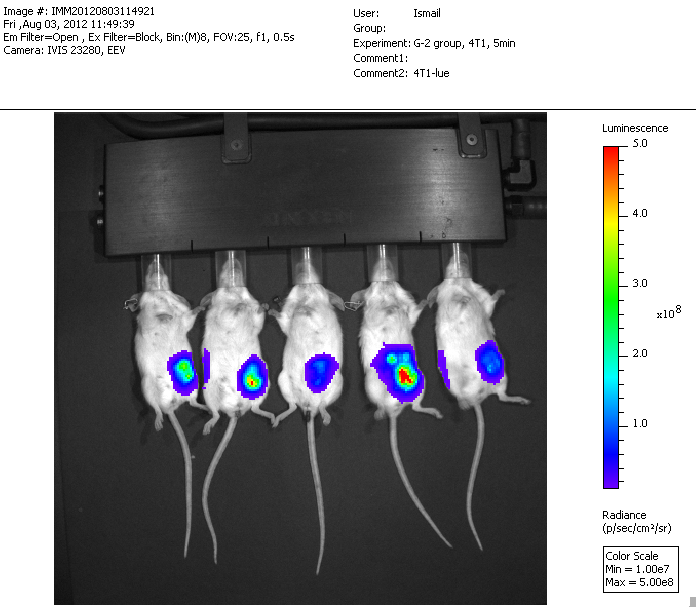

Supplement: S1 File — (ZIP) [file pone.0314577.s001.zip › S1 File/Fig 5D/14 PBS.tif]

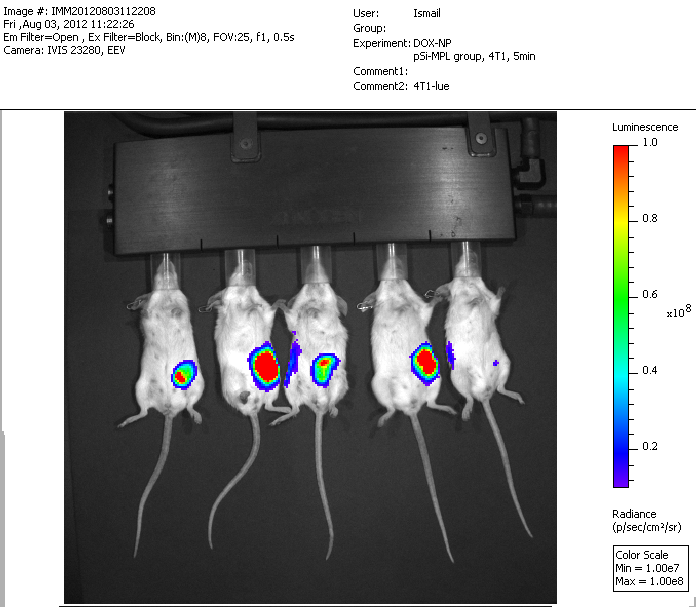

Supplement: S1 File — (ZIP) [file pone.0314577.s001.zip › S1 File/Fig 5D/14 Dox-NP+pSi-MPL.tif]

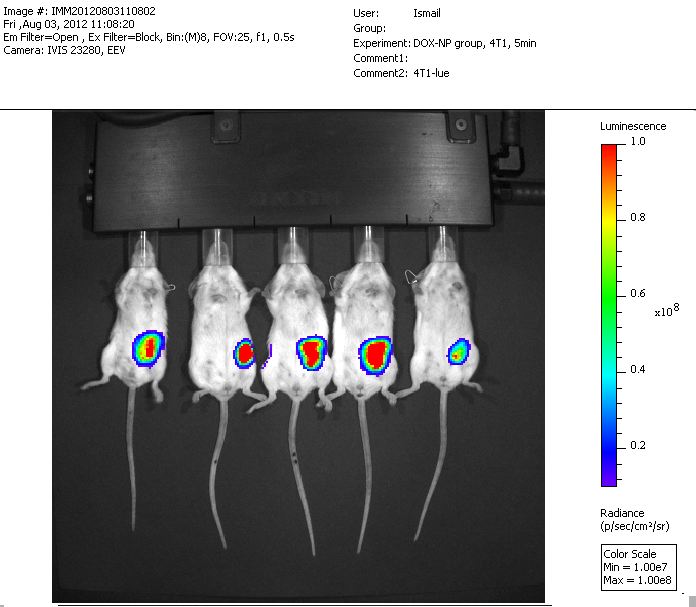

Supplement: S1 File — (ZIP) [file pone.0314577.s001.zip › S1 File/Fig 5D/14 Dox-NP.tif]

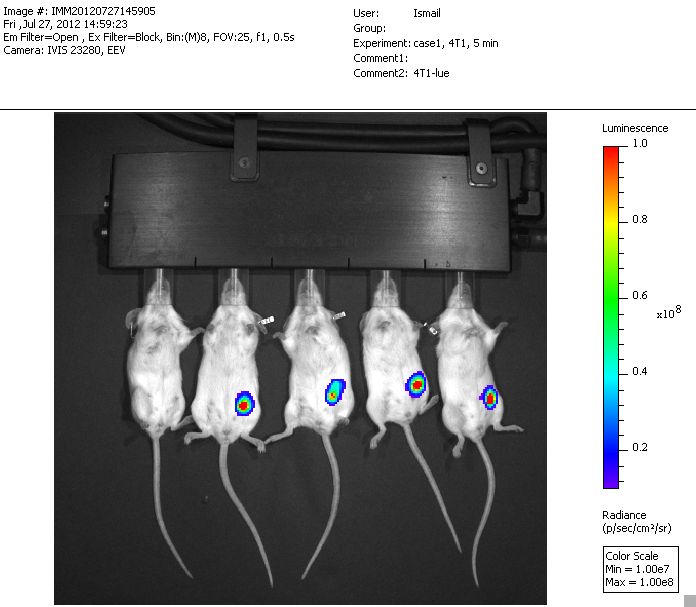

Supplement: S1 File — (ZIP) [file pone.0314577.s001.zip › S1 File/Fig 5D/7 PBS.tif]

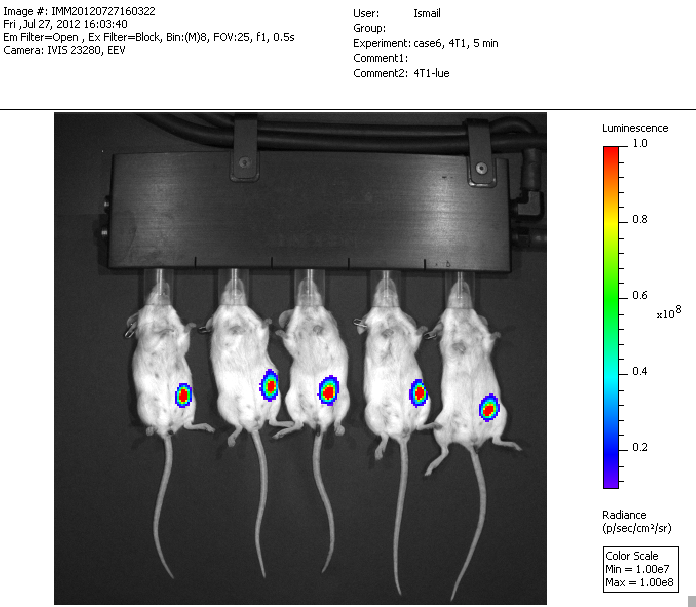

Supplement: S1 File — (ZIP) [file pone.0314577.s001.zip › S1 File/Fig 5D/7 Dox-NP.tif]

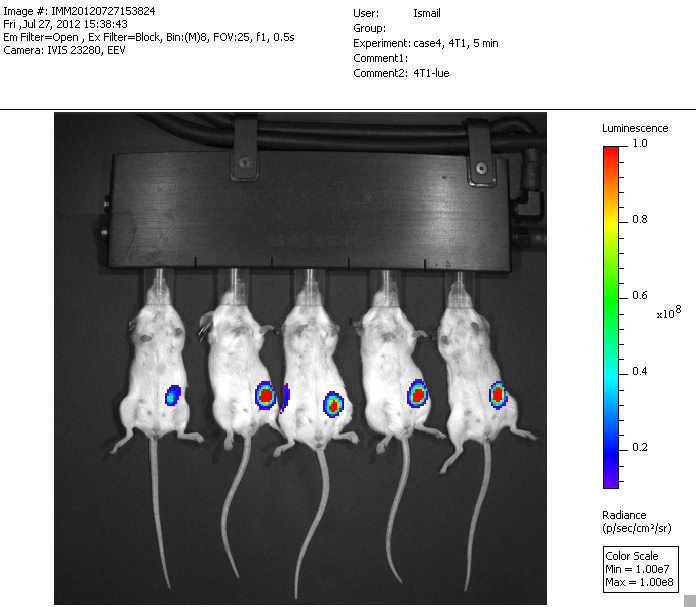

Supplement: S1 File — (ZIP) [file pone.0314577.s001.zip › S1 File/Fig 5D/7 Dox-NP+pSi-MPL.tif]

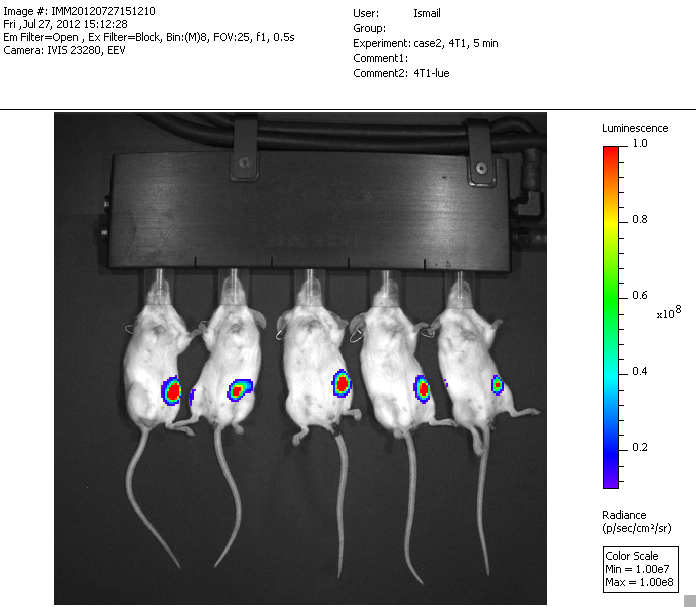

Supplement: S1 File — (ZIP) [file pone.0314577.s001.zip › S1 File/Fig 5D/7 pSi-MPL.tif]

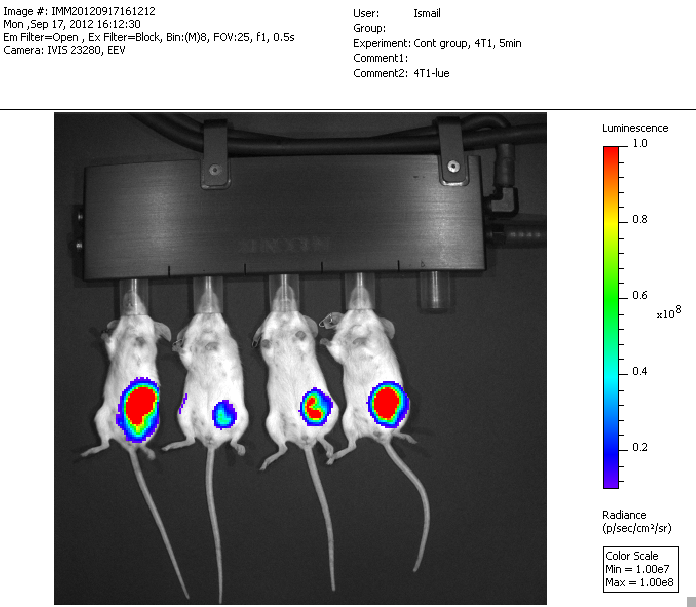

Supplement: S1 File — (ZIP) [file pone.0314577.s001.zip › S1 File/Fig 5H/17 PBS.tif]

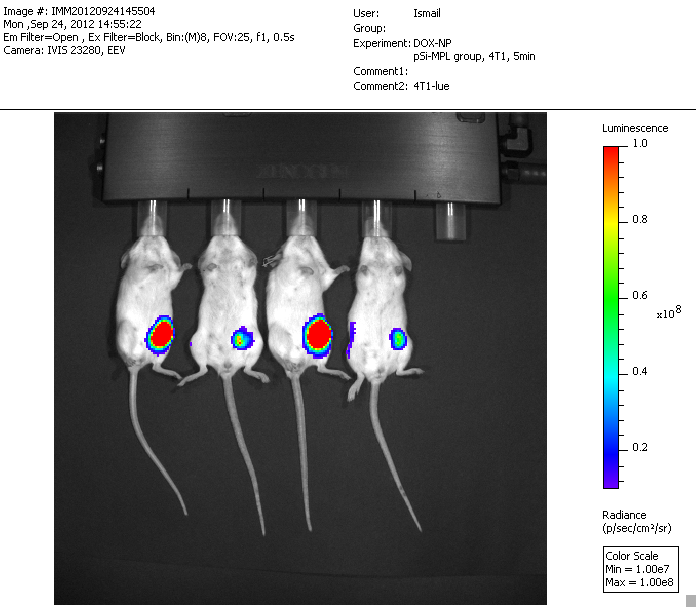

Supplement: S1 File — (ZIP) [file pone.0314577.s001.zip › S1 File/Fig 5H/24 DOX +pSi-MPL group.tif]

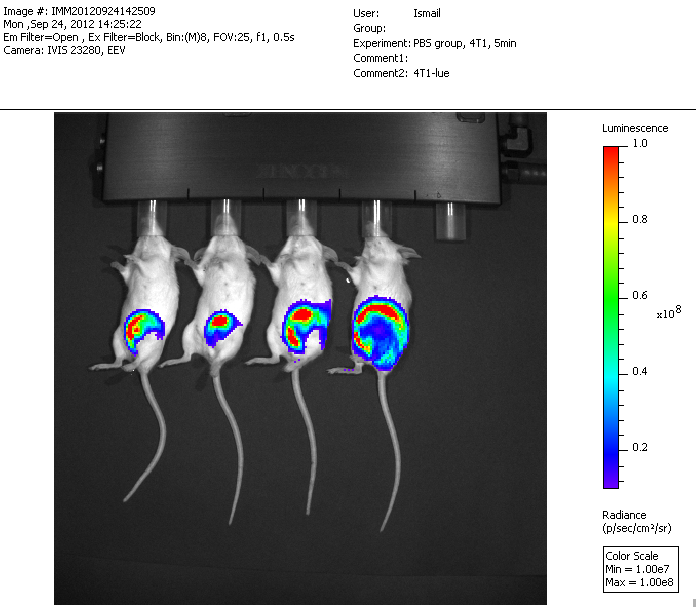

Supplement: S1 File — (ZIP) [file pone.0314577.s001.zip › S1 File/Fig 5H/24 PBS.tif]

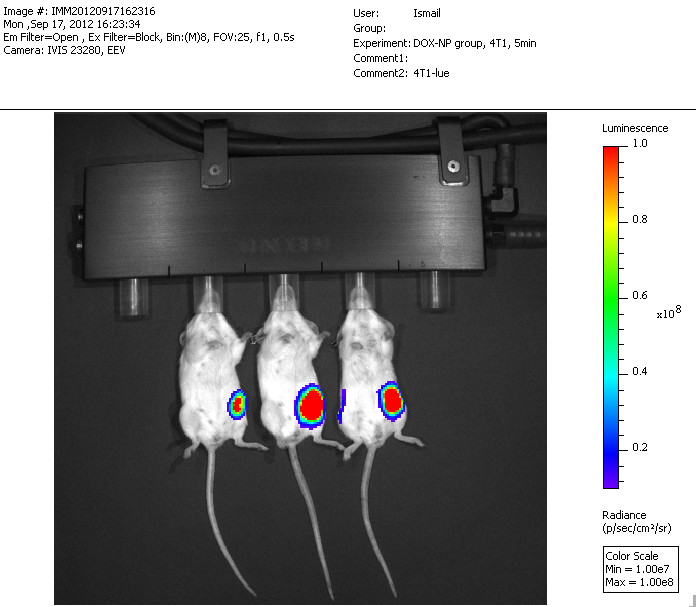

Supplement: S1 File — (ZIP) [file pone.0314577.s001.zip › S1 File/Fig 5H/17 DOX.tif]

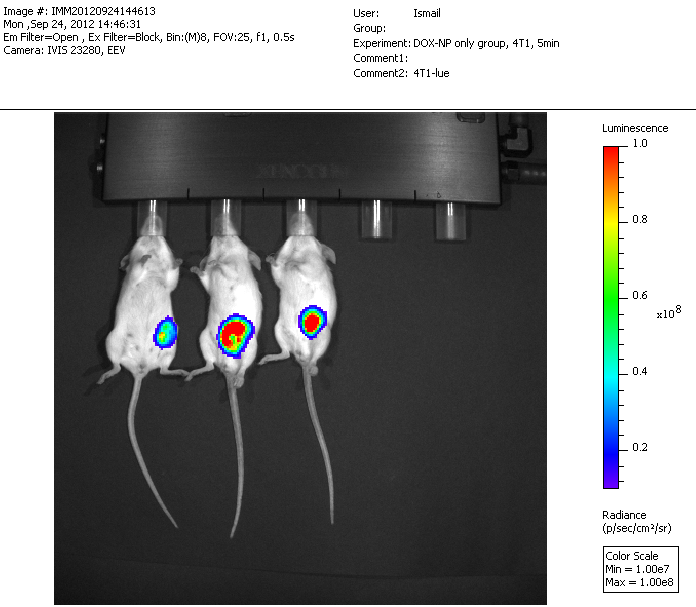

Supplement: S1 File — (ZIP) [file pone.0314577.s001.zip › S1 File/Fig 5H/24 DOX.tif]

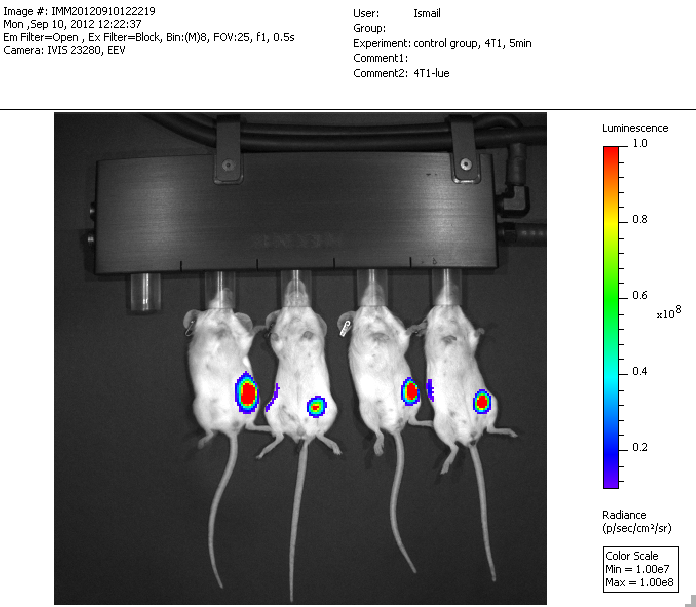

Supplement: S1 File — (ZIP) [file pone.0314577.s001.zip › S1 File/Fig 5H/10 PBS.tif]

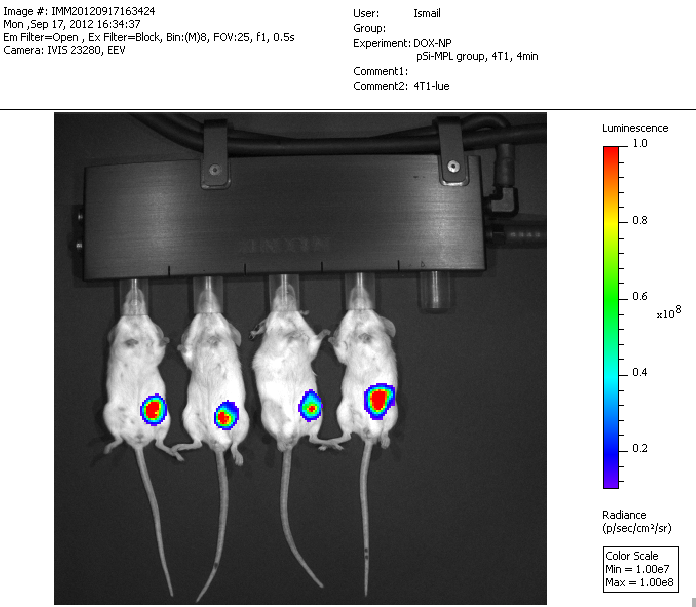

Supplement: S1 File — (ZIP) [file pone.0314577.s001.zip › S1 File/Fig 5H/17 DOX+pSi-MPL.tif]

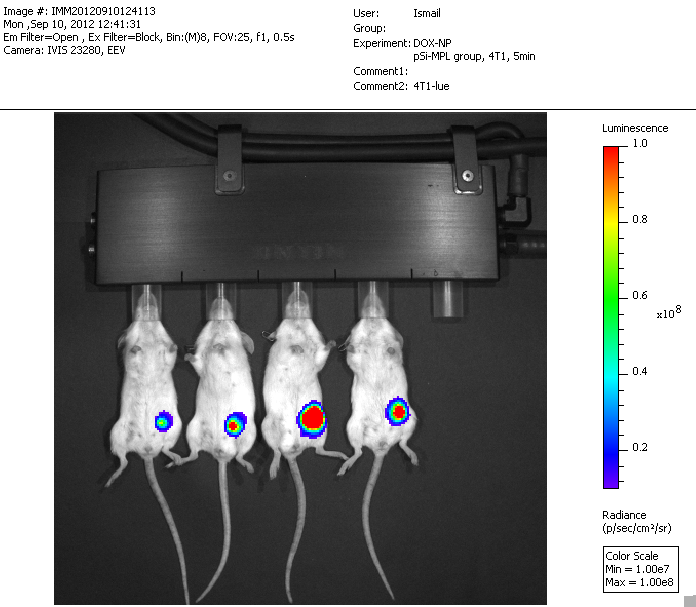

Supplement: S1 File — (ZIP) [file pone.0314577.s001.zip › S1 File/Fig 5H/10 DOX+pSi-MPL.tif]

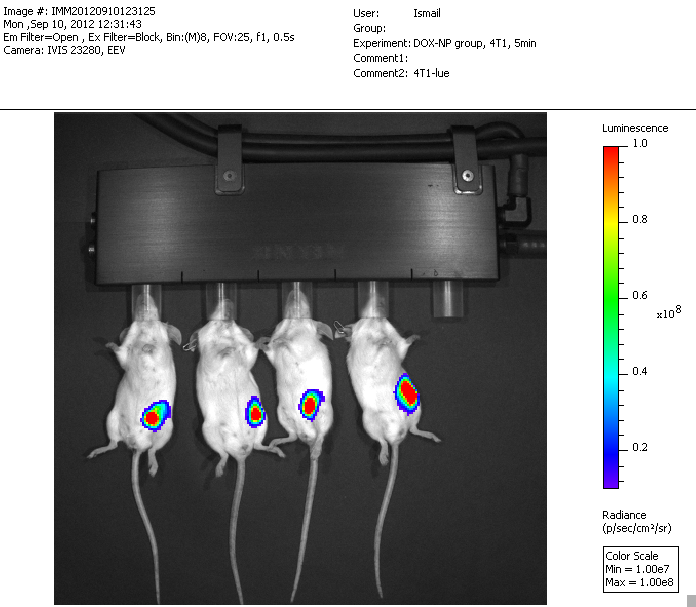

Supplement: S1 File — (ZIP) [file pone.0314577.s001.zip › S1 File/Fig 5H/10 DOX.tif]
